# Anterior cingulate cortex-related functional hyperconnectivity underlies sensory hypersensitivity in *Grin2b*-mutant mice

**DOI:** 10.1038/s41380-024-02572-y

**Published:** 2024-05-04

**Authors:** Soowon Lee, Won Beom Jung, Heera Moon, Geun Ho Im, Young Woo Noh, Wangyong Shin, Yong Gyu Kim, Jee Hyun Yi, Seok Jun Hong, Yongwhan Jung, Sunjoo Ahn, Seong-Gi Kim, Eunjoon Kim

**Affiliations:** 1 Graduate School of Medical Science and Engineering, Korea Advanced Institute of Science and Technology (KAIST), Daejeon, 34141 Korea; 2 Department of Anesthesiology and Pain Medicine, Seoul National University Bundang Hospital, Seongnam, 13620 Korea; 3 Center for Neuroscience Imaging Research, Institute for Basic Science (IBS), Suwon, 16419 Korea; 4 Emotion, Cognition & Behavior Research Group, Korea Brain Research Institute (KBRI), Daegu, 41062 Korea; 5 Department of Biological Sciences, Korea Advanced Institute of Science and Technology (KAIST), Daejeon, 34141 Korea; 6 Center for Synaptic Brain Dysfunctions, Institute for Basic Science (IBS), Daejeon, 34141 Korea; 7 Department of Biomedical Engineering, Sungkyunkwan University, Suwon, 16419 Korea; 8 Therapeutics and Biotechnology Division, Korea Research Institute of Chemical Technology (KRICT), Daejeon, 34114 Korea; 9 Department of Intelligent Precision Healthcare Convergence, Sungkyunkwan University, Suwon, 16419 Korea

**Keywords:** Neuroscience, Autism spectrum disorders

## Abstract

Sensory abnormalities are observed in ~90% of individuals with autism spectrum disorders (ASD), but the underlying mechanisms are poorly understood. GluN2B, an NMDA receptor subunit that regulates long-term depression and circuit refinement during brain development, has been strongly implicated in ASD, but whether *GRIN2B* mutations lead to sensory abnormalities remains unclear. Here, we report that *Grin2b*-mutant mice show behavioral sensory hypersensitivity and brain hyperconnectivity associated with the anterior cingulate cortex (ACC). *Grin2b*-mutant mice with a patient-derived C456Y mutation (*Grin2b*^C456Y/+^) show sensory hypersensitivity to mechanical, thermal, and electrical stimuli through supraspinal mechanisms. c-fos and functional magnetic resonance imaging indicate that the ACC is hyperactive and hyperconnected with other brain regions under baseline and stimulation conditions. ACC pyramidal neurons show increased excitatory synaptic transmission. Chemogenetic inhibition of ACC pyramidal neurons normalizes ACC hyperconnectivity and sensory hypersensitivity. These results suggest that GluN2B critically regulates ASD-related cortical connectivity and sensory brain functions.

## Introduction

Autism spectrum disorders (ASD), characterized by social deficits and repetitive behaviors, also involve sensory abnormalities in ~90% of cases, which could be causally associated with anxiety, social dysfunctions, and repetitive behaviors [1–6]. Increased sensory sensitivity in ASD would evoke enhanced pain perception and responses [7–10], but the underlying neural mechanisms are poorly understood. Previous studies on mouse models of ASD have provided valuable mechanistic insights into the sensory abnormalities [1, 11–20], which involve abnormalities in the peripheral and central nervous systems.

Examples of peripheral deficits include impaired presynaptic function in peripheral somatosensory neurons of *Mecp2* and *Gabarb3*-mutant mice [12, 16, 21], impaired TRPV1 function in dorsal root ganglia neurons of *Shank3*-mutant mice with impaired heat hyperalgesia [14], and impaired Kv1 potassium channel function in hyperexcitable dorsal root ganglia neurons of *Cntnap2*-mutant mice with sensory hypersensitivity [17]. Examples of central deficits include GABA neuronal deficits in the somatosensory cortex of *Shank3*-mutant mice with sensory hypersensitivity [11] and decreased excitatory synaptic transmission in somatosensory cortical pyramidal neurons of *Syngap1*-mutant mice with sensory hyposensitivity [13]. However, little is known about whether there are contributions from additional cortical areas, such as the pain-related anterior cingulate cortex (ACC), and relevant connectivity. In addition, the synaptic mechanisms underlying the sensory abnormalities remain largely unclear [13].

*GRIN2B* encodes the GluN2B subunit of NMDA receptors (NMDARs), and its mutations have been implicated in various brain disorders, including ASD, developmental delay, intellectual disability, epileptic encephalopathy, attention-deficit/hyperactivity disorder, schizophrenia, obsessive-compulsive disorder [22–37]. Functionally, GluN2B-containing NMDARs are known to regulate various aspects of brain development and functions by modulating excitatory synapse development and synaptic plasticity, including long-term depression (LTD) [38–41]. GluN2B mutations in mice lead to neurodevelopmental, synaptic, and learning and memory phenotypes, including neonatal lethality, suppressed LTD, and spatial/working memory deficits [42–47]. However, it remains unclear whether GluN2B dysfunction leads to abnormal neural connectivity and whether such impairments could be associated with the pathophysiology of GRIN2B-related brain disorders. More recently, we reported that mice carrying a patient-derived C456Y mutation [24] (*Grin2b*^C456Y/+^ mice) display suppressed LTD associated with anxiety-related abnormalities [48]. These phenotypes, observed at juvenile and adult stages, are improved by chronic pharmacological stimulation of NMDAR function during early postnatal stages [48], suggesting that early correction of NMDAR dysfunction has long-lasting effects.

In the present study, we show that *Grin2b*^C456Y/+^ mice display behavioral sensory hypersensitivities, which involve supraspinal mechanisms, ACC hyperactivity and hyperconnectivity, and enhanced ACC neuronal excitatory synaptic transmission. Chemogenetic suppression normalizes the hyperactivity and hyperconnectivity of the mutant ACC neurons and the behavioral hypersensitivity of the mutant mice. These results suggest that GluN2B critically regulates cortical connectivity and sensory brain functions.

## Methods

### Animals

The *Grin2b*^C456Y/+^ mice were as described previously [48]. To genotype these mice, the following primer set was used: forward, ACGACTCTTTGTGGAGGAGGG; reverse, CCATATCACAGCTTACTTCAATGT. For *Cdx2-Cre*;*Grin2b*^fl/+^ mice, *Grin2b*^fl/+^ mice with floxed *Grin2b* exon 5 were generated by Biocytogen. These mice were genotyped using the following primers: forward, GGTTTTGGGGGTCGGAGGAAGAAAT; reverse, CTCCCTGTCCTTTTGAGGGAGAAGC. The *Grin2b*^fl/+^ mice were crossed with *CDX2P-NLS-Cre* mice (JAX #009350), which express a nuclear-localized Cre recombinase under the control of a ~9.5-kb promoter and an upstream sequence element in the human CDX2 gene. The Cre transgene is expressed in all embryonic tissues caudal to the cervical spinal segment 2 during development [21, 49]. *Cdx2-Cre*;*Grin2b*^fl/+^ mice were genotyped using the following PCR primer sets: Cdx2-control-forward, CAAATGTTGCTTGTCTGGTG; Cdx2-control-reverse, GTCAGTCGAGTGCACAGTTT; Cdx2-mutant-forward, AGGAGCCAGCGGAGCAC; Cdx2-mutant-reverse, ACATGTCCATCAGGTTCTTGC. To selectively label ACC pyramidal neurons in *Grin2b*^C456Y/+^ mice, we generated *CaMKII*$\alpha$*-Cre*;Ai9;*Grin2b*^C456Y/+^ mice by crossing *CaMKII*$\alpha$*-Cre*;*Grin2b*^C456Y/+^ or *CaMKII*$\alpha$*-Cre*;*Grin2b*^+/+^ mice with Ai9 mice (B6.Cg-Gt(ROSA)26Sortm9(CAG-tdTomato)Hze/J, JAX#007909). The resulting triple-mutant mice were genotype using the following primers: Cre-forward, GATCTCCGGTATTGAAACTCCAAC; Cre-reverse: GCTAAACATGCTTCATCGTCGG. Mice were bred in-house and maintained with standard laboratory chow and water in ventilated cages in a temperature/humidity-controlled facility under a 12-h dark/light cycle according to the requirements of Animal Research at KAIST. All behavior, electrophysiology, and fMRI experiments were conducted using adult male mice aged 8 to 11 weeks. All procedures were approved by the Committees of Animal Research at KAIST (KA2020-90).

### Behavioral assays

All sensory behavioral experiments were conducted during the light phase of the circadian cycle unless stated otherwise. The investigators performing the experiments were blind to the mouse genotype. The mice were placed in the behavior room at least 30 min before the test to allow acclimation to the experimental apparatus. All behavioral experiments, except for those assessing anxiety behavior (i.e., the light–dark test or elevated plus maze test), were conducted under the lighting condition of ~100 lux. Additional information on behavioral tests is available in Supplementary Information.

### c-fos staining

Ninety min after entering the fear chamber with electric foot shocks (Experimental group) or without any electric foot shock (Control group), mouse brains were extracted, perfused with 4% paraformaldehyde, and post-fixed overnight. After post-fixation, brains were embedded into 3% phosphate-buffer saline (PBS)-agarose gel and serially sliced (50 μm) using vibratome (VT1200s, Leica). Slides were washed and blocked in 5% normal goat serum in PBS + 0.3% Triton X-100 for 1 h at room temperature and incubated in the primary antibody against c-fos (CS-2250s, Cell signaling) and NeuN (MAB377, Millipore) overnight at 4 °C. Primary antibodies were detected by incubating with the secondary antibody (anti-mouse Alexa488 for NeuN Ab, anti-rabbit Alexa 594 for c-fos Ab) at room temperature for 2 h. DAPI (4′,6-diamidino-2-phenylindole, 1:10,000) was co-administered with secondary antibodies. After secondary antibody staining, slices were mounted and imaged using a slide scanning microscope (Axio Scan.Z1). The number of c-fos and NeuN particles was automatically quantified using the AMaSiNe program [50].

### Electrophysiology

Ex-vivo electrophysiological experiments were performed to evaluate synaptic transmission and intrinsic excitability. Additional information on electrophysiology is available in Supplementary Information.

### Virus reagents

All viruses were purchased from Addgene. The pAAV-CaMKIIa-hM4D(Gi)-mCherry for CNO-induced neuronal silencing was a gift from Dr. Bryan Roth (Addgene viral prep #50476-AAV5), and the pAAV-CaMKIIa-mCherry was a gift from Dr. Karl Deisseroth (Addgene viral prep #114469-AAV5). Viruses were used for all chemogenetic experiments.

### Stereotactic surgeries

All stereotactic surgeries were conducted under aseptic condition and isoflurane-based anesthesia (4.5%, v/v; induction, 1%, v/v; maintenance). Viruses were injected into ACC or S1 bilaterally at appropriate stereotaxic coordinates [anterior-posterior (AP) and medial-lateral (ML) relative to bregma; dorsal-ventral (DV) relative to brain surface at target coordinate]; +0.8 mm AP, ±0.3 mm ML, –1.3 mm DV for ACC and –0.3 mm AP, ±1.9 mm ML, –0.9 mm DV for S1. Viruses were infused at a rate of 100 nl/min (total 500 nl) using NanoFil Needle (33G, blunt, NF33BL-2, WPI) connected to a Hamilton syringe by tubing backfilled with mineral oil. The needle was slowly removed 8 min after infusion.

### Chemogenetic rescue of sensory hypersensitivity

At 3–4 weeks after stereotactic surgery, the mice were subjected to behavioral experiments, such as the open field test, von Frey test, hot-plate test, thermal place-preference test, and electric foot-shock test. At 30 min before the behavioral tests, clozapine-N-oxide (CNO, 5 mg/kg) was injected intraperitoneally. After behavioral experiments, mouse brains were extracted, fixed in 4% paraformaldehyde, and assessed to confirm virus expression and targeting.

### Early chronic treatment of d-cycloserine

d-cycloserine/DCS (Sigma) was dissolved in 0.1% saccharin-based drinking water at the concentration of 80 mg/ml. DCS was orally administered at a dose of 40 mg/kg twice a day during postnatal days 7–16, as described previously [48].

### Functional MRI-animal preparation and stimulation

A total of 75 mice (~30 g, 8–11 weeks old, male) were used in four different studies: (1) 11 heterozygous mutant (HT, *Grin2b*^C456Y/+^) and 10 wild-type (WT) mice for whisker stimulation-evoked fMRI to determine which brain regions exhibit abnormal functional activity in the mutant mice, (2) 11 HT and 11 WT mice with chemogenetic viral transfection for whisker stimulation-evoked fMRI before and after intraperitoneal injection of CNO dissolved in DMSO to investigate the effects of chemogenetic silencing of ACC, (3) 7 HT and 7 WT mice with chemogenetic viral transfection for whisker stimulation-evoked fMRI before and after DMSO injection as control for the CNO effects, and (4) 9 HT and 9 WT mice for whisker stimulation-evoked fMRI before and after CNO injection solely to examine the effect of chemogenetic actuators. For somatosensory activation, the electrical stimulation was given to the right whisker pad at 4 Hz with a pulse width of 0.5 ms and a current intensity of 0.9 mA (performed using Master 9; World Precision Instruments) via a customized square electrode pad (2 × 2 mm^2^), which has two anodes and one cathode.

The animals were initially induced with 2–4% isoflurane. Once induced, each mouse was carefully positioned on a customized cradle equipped with two ear plugs, a bite bar, and a mask for the duration of the experiment. Following the completion of the experimental setup, isoflurane administration was discontinued. All MRI experiments were conducted under anesthesia, which was maintained by continuous intravenous infusion of dexmedetomidine (0.05 mg/kg/h) through the tail vein with 0.3% isoflurane, while allowing the mice to self-breathe [51]. During fMRI experiments, heart rate and motion-sensitive respiration signals were continuously measured using a physiological monitoring system (Model 1030, Small Animal Instruments Inc) along with body temperature being maintained at 37 ± 0.5 ^o^C with a warm-water heating system via a rectal thermometer.

### Functional MRI-MRI experiments

All MRI experiments were performed on a 15.2 Tesla MRI system (Bruker BioSpec) with an actively shielded 6-cm diameter gradient operating with a maximum strength of 100 G/cm and a rise time of 110 μs. The animal brain was positioned as close to the iso-center of the magnet, and a 15-mm inner diameter (ID) customized surface coil used for RF transmission and reception was centered over the imaging volume of interest. The field homogeneity was optimized using both global and local shimming on the ellipsoidal volume covering the cerebrum (MAPSHIM, ParaVision 6, Bruker BioSpin). Anatomical images were acquired using the fast low angle shot magnetic resonance imaging (FLASH) sequence with the following parameters: repetition time (TR)/ echo time (TE) = 384/3.3 ms, flip angle (FA) = 45°, field of view (FOV) = 15.8 × 7.9 mm^2^, matrix = 256 × 128, in-plane resolution = 62 × 62 μm^2^, slice thickness = 500 μm, contiguous 20 coronal slices without gap, and number of excitations (NEX) = 8. These anatomical images were used for the spatial normalization to a common brain space. Functional data were acquired using single-shot gradient echo (GE) echo planar imaging (EPI) sequence with the following parameters: TR/TE = 1000/11.5 ms, FA = 50°, FOV = 15.8 × 7.65 mm^2^, matrix = 120 × 58, in-plane resolution = 132 × 132 μm^2^, slice thickness = 500 μm, and contiguous 20 coronal slices without gap.

In each mouse, somatosensory-evoked fMRI and resting-state (rs)-fMRI were acquired. Each somatosensory-evoked fMRI trial consisted of a 30-s pre-stimulus, 20-s stimulus, 30-s inter-stimulus, 20-s another stimulus, and finally 30-s post-stimulus period. For fMRI of mice with chemogenetic actuators, somatosensory-evoked fMRI was conducted before and 10 min after intraperitoneal injection of CNO or DMSO. The duration of fMRI scans after injection of CNO or DMSO was less than 1.5 h. For the signal averaging, 10 to 15 fMRI trials were performed for each stimulus condition. Following the evoked fMRI scans, an additional 10-min rs-fMRI scan was obtained in some animals.

### Functional MRI-data analysis

All MRI data were analyzed using the Analysis of Functional Neuroimages package (AFNI), FMRIB Software Library (FSL), Advanced Normalization Tools (ANTs), and Matlab codes (The Mathworks).

#### Somatosensory-evoked fMRI

The fMRI activation maps for individual animals were generated using preprocessing and general linear model (GLM) analysis. The fMRI data processing was previously described in detail [52, 53]. First, the preprocessing pipeline in functional data included slice timing correction, image realignment for minor head motions during intra- and inter-scans, linear signal detrending for drift removal, and time-course normalization by the average of the baseline period. All repeated fMRI trials per stimulus condition were averaged to improve the signal to noise ratio. Second, functional data was co-registered to an anatomic image, and then spatially normalized onto the Allen moue brain atlas using deformation parameters obtained between anatomic image and mouse brain atlas. Third, the functional data normalized in a common space were smoothed with a Gaussian filter of 0.2 mm full width at half maximum (FWHM). The fMRI maps of the individual animal were calculated by a GLM analysis using a stimulus paradigm convolved with dexmedetomidine/isoflurane specific-hemodynamic response function [51]. The group-averaged maps were obtained by one sample *t*-test using non-parametric permutation with a significance at threshold-free cluster enhancement (TFCE)-corrected *p* < 0.01. To evaluate how functional activations differed between HT and WT mice, Student’s *t*-test was also conducted at a significance level of TFCE-corrected *p* < 0.01.

#### Quantitative ROI analysis

To estimate the regional specificity of sensory-evoked fMRI responses between HT and WT mice, ten different regions of interest (ROIs) corresponding to somatosensory active sites were defined on Allen mouse brain atlas: anterior cingulate cortex (ACC), primary and secondary motor cortex (M1, M2), primary somatosensory cortex of barrel field (S1BF), secondary somatosensory cortex (S2), ventral posterior thalamic nucleus (VP), posterior medial thalamic nucleus (PO), superior colliculus motor-related (SCm) and periaqueductal gray (PAG). The ROIs for ACC and PAG, which are centrally located in brain structure, include areas within both hemispheres. The S1BF ROIs were separated into contralateral and ipsilateral hemisphere to the stimulated whisker pad (cS1BF, iS1BF), whereas the other ROIs were defined as the contralateral region to the stimulus side.

For quantification, time courses were extracted from each ROI, and then percent signal changes over the stimulus duration were averaged. To compare functional signal changes between HT and WT mouse groups under the same experimental condition, a Student’s *t*-test was performed. Additionally, to identify the most aberrant region in *Grin2b*^C456Y/+^ mice, the regions with statistically significant differences were used to apply the minimum redundancy maximum relevance (mRMR) feature selection method [54] using the “fscmmr” function in Matlab. At each ROI, we assessed the relevance between percent changes of HT and WT groups and redundancy against other ROIs using mutual information. Subsequently, the importance of each ROI was scored by calculating the quotient of these relevance and redundancy measures. This methodology enabled us to rank ROIs for the classification of the two groups, selecting the region with the highest correlation between HT and WT groups while minimizing correlation with other ROIs. This fMRI-guided group classification was used to determine the target region for subsequent chemogenetic modulation. ROI-wise sensory stimulus-evoked fMRI signals before and after chemogenetic modulation were compared using a paired *t*-test.

#### Connectivity analysis

To investigate whether brain functional connectivity patterns are intrinsically aberrant in HT mice, we first classified functional resting-state networks (RSNs) using independent component analysis (ICA) decomposition. Preprocessing steps for rs-fMRI data were similar to those for evoked-fMRI analysis, including slice timing correction, image realignment, linear signal detrending, voxel-wise time course normalization, and spatial smoothing with a Gaussian kernel of 0.2 mm FWHM. To enhance the reliability of functional connectivity, three additional preprocessing steps were involved: de-spiking fMRI time courses, clearing data by regressing out 12 motion confounds (6 parameters + temporal derivatives), and bandpass filtering (0.01 < f < 0.3 Hz). In a common space, temporally concatenated group ICA was performed to infer RSNs using the Multivariate Exploratory Linear Decomposition into Independent Components (MELODIC), a part of FSL. The spatial dimensionality of the ICA decomposition was set to 20, with a threshold level of 0.5 for the alternative hypothesis tests. From the ICA-derived spatial maps, a total of 14 components were visually identified as plausible group-level RSNs based on a mouse brain atlas. The identified 14 networks mainly included the following areas: S1 of mouth and partial S2 (S1M + S2), retrosplenial cortex (RSC), S1BF, S1 of forelimb and partial S2 (S1FL + S2), M1 + M2, striatum dorsal region (STRd), visual cortex (VIS), ACC, hippocampus (HIP), auditory cortex and temporal association cortex (AUD+TEa), right superior colliculus sensory-related (Rt.SCs), left superior colliculus sensory-related (Lt.SCs), SCm, and hypothalamus (HY).

Time courses from each RSN were extracted from rsfMRI and evoked fMRI data in each animal using dual regression. Subsequently, functional connectivity matrices were constructed using Pearson correlation (r) for all pairs of 14 components in each experimental condition to compare the functional connectivity between HT and WT mice. The statistical difference in correlation z scores from Fisher’s r-to-z transformation for each pair between HT and WT mice was determined using a Student’s *t*-test.

### Statistical analysis

Statistical analyses were performed using Prism GraphPad 9. Data with non-parametric distribution were analyzed by the Mann–Whitney test, and those with parametric distribution were analyzed by the Student’s *t*-test. If data were parametric but showed a significant difference in variance on the F-test, Welch’s correction was used. Multiple groups were compared using two-way analysis of variance with Bonferroni post-hoc test.

## Results

### *Grin2b*^C456Y/+^ mice display somatosensory hypersensitivity through supraspinal mechanisms

To determine whether *Grin2b*^C456Y/+^ mice carrying the human C456Y mutation [24, 48] display somatosensory functional alterations, we subjected the mice to various sensory behavioral tests, including electronic von Frey, thermal place-preference, hot-plate, and electric foot-shock tests.

**Fig. 1:**
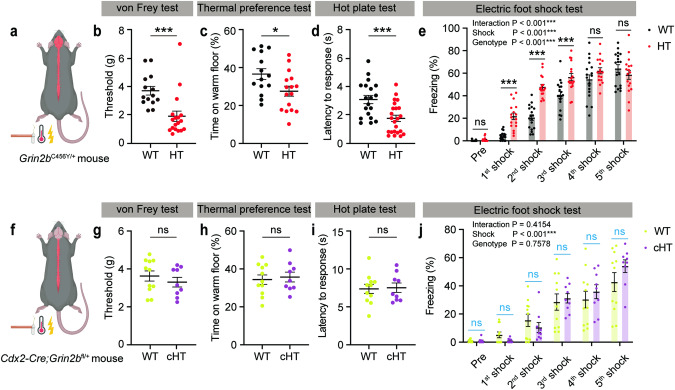
*Grin2b*^C456Y/+^ mice display somatosensory hypersensitivity to mechanical, thermal, and electric stimuli through supraspinal mechanisms. **a** A schematic diagram showing the *Grin2b*^C456Y/+^ mutation in the whole nervous system, including the brain and spinal cord. The three tested types of somatosensory stimuli (mechanical, thermal, and electric) are indicated. **b** Increased sensitivity to mechanical stimuli in *Grin2b*^C456Y/+^ mice (2–3 months) in the electronic von Frey test, as shown by threshold of response. Statistical details are presented in Supplementary Table 1 (*n* = 14 mice [WT], 18 [HT/*Grin2b*^C456Y/+^], Mann–Whitney test). **c** Increased sensitivity to thermal stimuli in *Grin2b*^C456Y/+^ mice (2–3 months) in the thermal place-preference test, as shown by time spent on a warm floor (*n* = 14 [WT], 16 [HT], Student’s *t*-test). **d** Increased sensitivity to thermal stimuli in *Grin2b*^C456Y/+^ mice (2–4 months) in the hot-plate test, as shown by latency to withdrawal response (*n* = 19 [WT], 23 [HT], Student’s t-test). **e** Increased sensitivity to electric stimuli in *Grin2b*^C456Y/+^ mice (2–3 months) in the electric foot-shock test, as shown by freezing levels during repeated stimulations (*n* = 18 [WT],17 [HT], two-way ANOVA with Sidak’s test). **f** A schematic diagram showing the *Cdx2-Cre;Grin2b*^fl/+^ mutation in peripheral neural regions caudal to the second cervical spinal segment. **g** Normal sensitivity to mechanical stimuli in *Cdx2-Cre;Grin2b*^fl/+^ mice (2–3 months) in the electronic von Frey test, as shown by response threshold (*n* = 11 [WT], 9 [conditional-HT/cHT], Student’s t-test). **h** Normal sensitivity to thermal stimuli in *Cdx2-Cre;Grin2b*^fl/+^ mice (2–3 months) in the thermal place-preference test, as shown by warm-floor time. (*n* = 11 [WT], 9 [cHT], Student’s t-test). **i** Normal sensitivity to thermal stimuli in *Cdx2-Cre;Grin2b*^fl/+^ mice (2–3 months) in the hot-plate test, as shown by response latency (*n* = 11 [WT], 9 [cHT], Student’s t-test). **j** Normal sensitivity to electric stimuli in *Grin2b*^C456Y/+^ mice (2–3 months) in the electric foot-shock test, as shown by freezing levels during repeated stimulations. (*n* = 11 [WT], 9 [cHT], two-way ANOVA, Student’s t-test [multiple comparisons; indicated in blue]). Significance is indicated as * (<0.05), ** (<0.01), *** (<0.001), or ns (not significant).

*Grin2b*^C456Y/+^ mice exhibited a lower threshold for mechanical stimuli in the electronic von Frey test, as compared with WT mice (Fig. 1a, b). *Grin2b*^C456Y/+^ mice also displayed thermal hypersensitivity, as shown by the decreased preference for a warm floor in the thermal place-preference test and decreased latency to withdrawal response in the hot-plate test (Fig. 1c, d). Lastly, *Grin2b*^C456Y/+^ mice displayed increased sensitivity to electric foot shocks, as shown by increased freezing responses during repeated foot shocks (Fig. 1e). These results suggest that *Grin2b*^C456Y/+^ mice are hypersensitive to mechanical, thermal, and electrical aversive somatosensory stimuli.

Given that *Grin2b* is expressed both in the brain and in non-brain peripheral neural tissues (spinal cord and peripheral neurons) [38, 55, 56], we restricted *Grin2b* deletion to non-brain neural tissues caudal to the cervical 2 spinal segment using the *Cdx2-Cre* mouse line (Fig. 1f; Supplementary Fig. 1a, b) [21, 49]. Interestingly, *Cdx2-Cre;Grin2b*^fl/+^ mice (exon 5 deletion) did not show altered behavioral sensitivity to mechanical, thermal, or electric stimuli (Fig. 1g–j). Furthermore, *Cdx2-Cre;Grin2b*^fl/+^ mice showed largely normal locomotor and anxiety-like behavior, except for a mild decrease in open-field locomotion (Supplementary Fig. 1c–k). These results suggest that *Grin2b* haploinsufficiency leads to somatosensory hypersensitivity through supraspinal mechanisms.

### *Grin2b*^C456Y/+^ brain regions show stronger responses to sensory stimuli

**Fig. 2:**
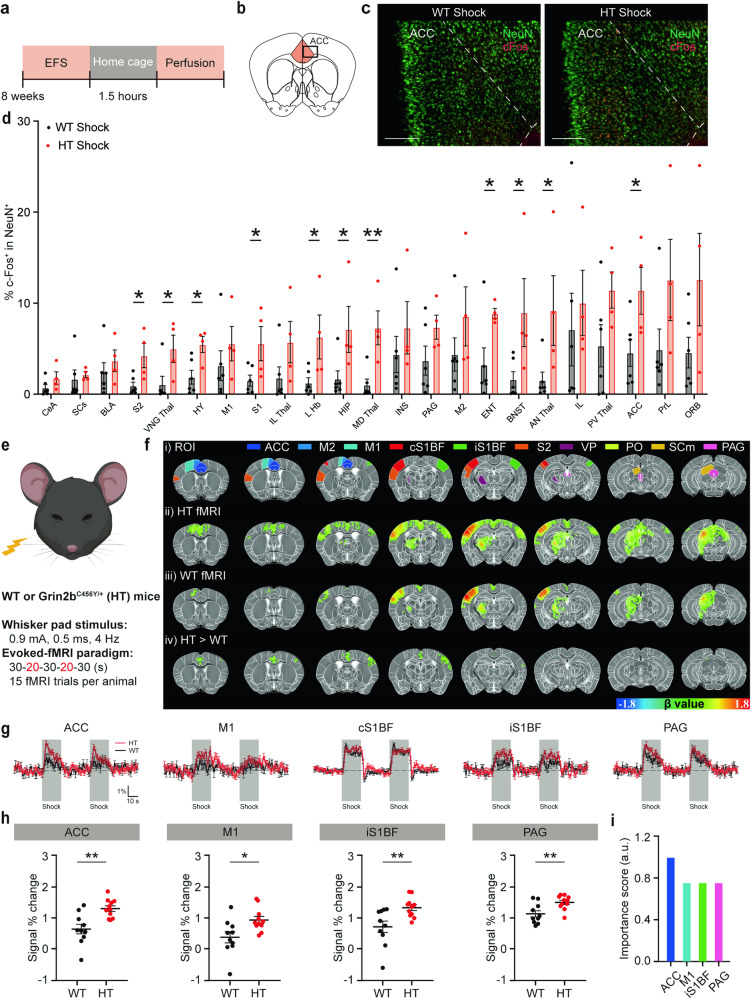
*Grin2b*^C456Y/+^ brain regions display stronger increases in foot shock-induced c-fos signals and whisker stimulation-induced fMRI signals. **a** A schematic diagram showing electric foot shocks followed by home-cage rest (1.5 h) and brain perfusion for c-fos staining. **b**, **c** Anterior cingulate cortex/ACC as an example of a brain region showing stronger foot shock-induced increases in c-fos signals for *Grin2b*^C456Y/+^ mice relative to WT mice. NeuN staining was performed to identify c-fos signals in neurons. Scale bar, 200 µm. **d** Quantification of the results shown in Supplementary Fig. 2 indicating stronger foot shock-induced increases in c-fos signals in *Grin2b*^C456Y/+^ brain regions, as compared with those in WT mice. ACC anterior cingulate cortex, AN Thal anterior group of the dorsal thalamus, BLA basolateral amygdala, BNST bed nuclei of the stria terminalis, CeA central amygdala, ENT entorhinal cortex, HIP hippocampus, HY hypothalamus, IL infralimbic cortex, IL Thal intralaminar nuclei of the dorsal thalamus, INS insular cortex, L Hb lateral habenula, MD Thal medial group of the dorsal thalamus, M1 primary motor cortex, M2 secondary motor cortex, ORB orbital cortex, PAG periaqueductal gray, PrL prelimbic cortex, PV Thal paraventricular nucleus of the thalamus, SCs superior colliculus, S1 primary somatosensory cortex; S2, secondary somatosensory cortex; VNG Thal, ventral group of the dorsal thalamus (*n* = 6 mice [WT], 4 [HT], Student’s t-test). **e** A schematic diagram showing whisker-pad stimulation during fMRI imaging experiments. An anesthetized mouse (maintained by continuous intravenous infusion of dexmedetomidine with 0.3% isoflurane) was stimulated by a whisker pad shock (0.9 mA; 0.5 ms; 4 Hz; two 20-s stimulations interleaved with three 30-s intervals) while acquiring fMRI images at an ultrahigh field of 15.2 T. **f** fMRI activation maps and regions of interest (ROIs). fMRI signals were elicited by whisker-stimulation in *Grin2b*^C456Y/+^ and WT mice (2–3 months). ROIs in the first row indicate those used for fMRI signal quantifications in rows ii, iii, and iv. The signals in the last row (HT [*Grin2b*^C456Y/+^] > WT) indicate the numerical difference between β values of significantly different HT (*Grin2b*^C456Y/+^) and WT voxels, shown in upper rows (Student’s t-test). ACC, anterior cingulate cortex; M1, primary motor cortex; M2, secondary motor cortex; PAG, periaqueductal gray; PO, posterior complex of the thalamus; SCm, superior colliculus, motor related; cS1BF, contralateral primary somatosensory cortex of barrel field; iS1BF, ipsilateral primary somatosensory cortex of barrel field; S2, secondary somatosensory cortex; VP, ventral posterior complex of the thalamus. **g** Traces of fMRI signals before/during (gray blocks)/after whisker stimulation in different brain regions of *Grin2b*^C456Y/+^ and WT mice (2–3 months); see also Supplementary Fig. 4 for other brain regions. **h** Quantification of the results in (**g**); only significantly different results are shown (*n* = 11 mice [HT], 10 [WT], Student’s t-test). **i** Comparison of the importance scores in select brains, including the ACC (see “Methods” for details). Significance is indicated as * (<0.05), ** (<0.01), *** (<0.001), or ns (not significant).

To determine brain regions that abnormally respond to sensory stimuli in *Grin2b*^C456Y/+^ mice, we combined c-fos imaging analyses with electric foot shocks (Fig. 2a). The results, which were quantified using the AMaSiNe program [50], indicated that foot shock-induced c-fos signals were increased in various brain regions of *Grin2b*^C456Y/+^ mice compared with WT mice (Fig. 2b–d; Supplementary Fig. 2).

The regions exhibiting significant changes included the ACC, which is a pain-related brain region [57–59], as well as the somatosensory cortex, thalamus, hypothalamus, hippocampus, habenula, and bed nucleus of the stria terminalis (Fig. 2d). In contrast, baseline c-fos signals were largely comparable in WT and *Grin2b*^C456Y/+^ brain regions (Supplementary Fig. 3).

We also performed functional magnetic resonance imaging (fMRI), which enables real-time and repetitive monitoring of sensory stimulus-induced neuronal activities (Fig. 2e). Here, we used electrical whisker stimulation, which elicits stronger fMRI signals relative to those induced by electric foot shocks. We performed unilateral stimulation and used the contralateral side as control. Electrical whisker stimulation induced stronger increases in fMRI signals in multiple brain regions of *Grin2b*^C456Y/+^ mice compared with WT mice (Fig. 2f). Such brain regions included the ACC, M1, and PAG (Fig. 2g, h; Supplementary Fig. 4). Notably, a significant increase was observed in the ipsilateral but not contralateral primary sensory cortex, suggestive of the possibility of abnormal ipsilateral hypersensitivity. Among the four brain regions with altered signals, the ACC displayed the most significant difference between mutant and WT mice (ACC, *p* = 0.00149; PAG, *p* = 0.00665; iS1BF, *p* = 0.00847; M1, *p* = 0.01554) and had the highest importance score (see Methods for details) (Fig. 2i). These results collectively suggest that *Grin2b* haploinsufficiency in mice leads to neuronal hyperactivity especially in the ACC.

### Increased excitatory synaptic transmission in *Grin2b*^C456Y/+^ ACC pyramidal neurons

To determine the synaptic basis for the increased neuronal activity in *Grin2b*^C456Y/+^ neurons, we determined spontaneous and evoked synaptic transmissions in ACC neurons, which are known to display strong sensory stimulus-dependent neuronal and synaptic changes [57–59].

**Fig. 3:**
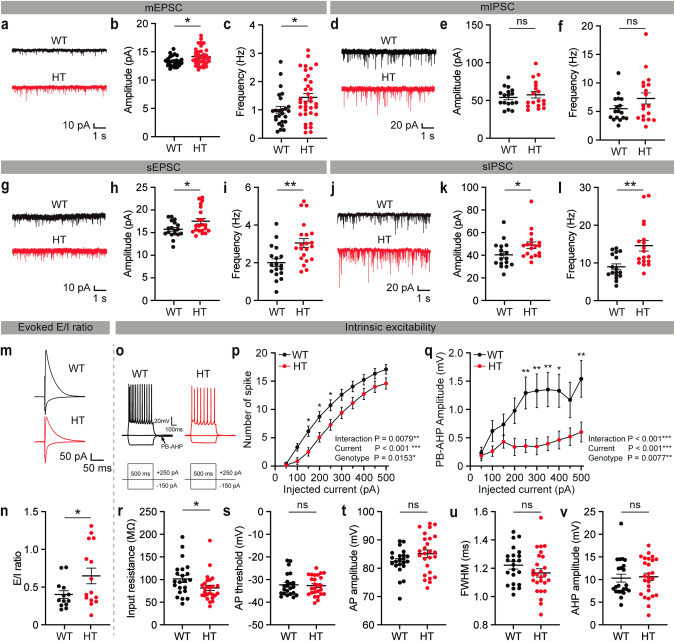
Increased excitatory synaptic transmission in *Grin2b*^C456Y/+^ ACC pyramidal neurons. **a**–**c** Increased frequency and amplitude of mEPSCs in *Grin2b*^C456Y/+^ layer 2/3 ACC pyramidal neurons (2–3 months) (*n* = 25 neurons from 4 mice [WT], 35, 4 [HT], Student’s t-test). **d**–**f** Normal frequency and amplitude of mIPSCs in *Grin2b*^C456Y/+^ layer 2/3 ACC pyramidal neurons (2–3 months) (*n* = 17, 4 [WT], 18, 4 [HT], Welch’s test [frequency], Student’s t-test [amplitude]). **g**–**i** Increased frequency and amplitude of sEPSCs in *Grin2b*^C456Y/+^ layer 2/3 ACC pyramidal neurons (2–3 months) (*n* = 18, 4 [WT], 21, 4 [HT], Student’s t-test). **j**–**l** Increased frequency and amplitude of sIPSCs in *Grin2b*^C456Y/+^ layer 2/3 ACC pyramidal neurons (2–3 months) (*n* = 17, 4 [WT], 18, 3 [HT], Mann–Whitney test [frequency], Student’s t-test [amplitude]). **m**, **n** Increased ratio of the eEPSCs and eIPSCs in *Grin2b*^C456Y/+^ layer 2/3 ACC pyramidal neurons (2–3 months) (*n* = 13, 3 [WT], 15, 4 [HT], Welch’s t-test). **o**–**v** Moderately increased excitability in *Grin2b*^C456Y/+^ layer 2/3 ACC pyramidal neurons (2–3 months), as supported by current-firing curve, post-burst afterhyperpolarization (PB-AHP), and input resistance, although not by action potential (AP) threshold, AP amplitude, AP half-width (full-width at half-maximum), and AHP amplitude after the first AP. These parameters were measured in the presence of pharmacological blockade of NMDA, AMPA, and GABA receptor currents (see “Methods” for details) (*n* = 22, 4 [WT], 26, 4 [HT], two-way ANOVA with Sidak’s test [current-firing curve and PH-AHP], Student’s t-test [input resistance, AP threshold/amplitude/half-width, AHP amplitude]). Significance is indicated as * (<0.05), ** (<0.01), *** (<0.001), or ns not significant.

Spontaneous excitatory synaptic transmission was increased in *Grin2b*^C456Y/+^ layer 2/3 ACC pyramidal neurons, compared with WT neurons, as shown by the frequency and amplitude of miniature excitatory postsynaptic currents (mEPSCs) (Fig. 3a–c). In contrast, miniature inhibitory postsynaptic currents (mIPSCs) were unchanged in *Grin2b*^C456Y/+^ neurons (Fig. 3d–f).

Spontaneous excitatory synaptic transmission in the presence of network activity (spontaneous EPSCs or sEPSCs), which is monitored by omitting action potential (AP)-blocking tetrodotoxin, was similarly increased in *Grin2b*^C456Y/+^ neurons (Fig. 3g–i), suggesting that network activity does not affect mEPSCs. In contrast, spontaneous inhibitory postsynaptic currents (sIPSCs) were increased in *Grin2b*^C456Y/+^ neurons (Fig. 3j–l), suggesting that network activity increases inhibitory synaptic transmission in the mutant brain.

Importantly, the ratio of evoked excitatory to inhibitory synaptic transmissions (eEPSCs/eIPSCs) was increased in *Grin2b*^C456Y/+^ layer 2/3 ACC pyramidal neurons (Fig. 3m, n). Parameters of intrinsic excitability showed mixed changes in *Grin2b*^C456Y/+^ neurons, as supported by opposing changes of related parameters (decreased current-firing curve and input resistance vs. decreased post-burst afterhyperpolarization) and unaltered action potential (AP)-related parameters (threshold, amplitude, half-width) and afterhyperpolarization after 1^st^ AP (Fig. 3o–v).

These results suggest that *Grin2b* haploinsufficiency increases excitatory synaptic transmission and induces mixed changes in neuronal excitability in *Grin2b*^C456Y/+^ ACC layer 2/3 pyramidal neurons.

### Chemogenetic ACC inhibition normalizes sensory hypersensitivity in *Grin2b*^C456Y/+^ mice

**Fig. 4:**
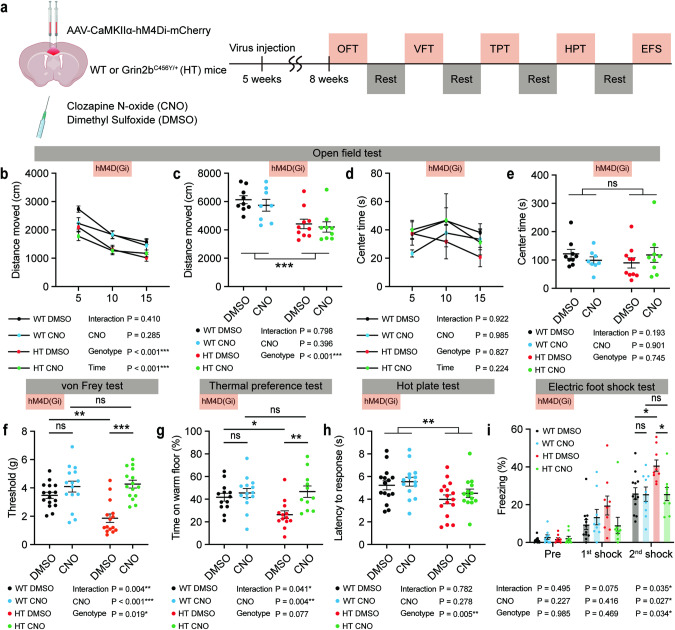
Chemogenetic ACC inhibition normalizes sensory hypersensitivity in *Grin2b*^C456Y/+^ mice. **a** Schema of chemogenetic modulation using DREADD. Injection of AAV-CaMKIIα-hM4Di-mCherry into the WT and *Grin2b*^C456Y/+^ ACC was followed by locomotor and sensory tests with CNO/DMSO treatment. **b**–**e** Chemogenetic inhibition of ACC neurons does not alter the open-field locomotion and anxiety-like behavior (center time) of *Grin2b*^C456Y/+^ mice (2–3 months) (*n* = 9 mice [WT-DMSO], 8 [WT-CNO], 10 [HT-DMSO], 9 [HT-CNO], three-way ANOVA [distance moved/center time], two-way ANOVA [total distance moved/center time]). **f**–**i** Chemogenetic inhibition of ACC neurons normalizes the sensory hypersensitivity of *Grin2b*^C456Y/+^ mice (2–3 months) in the electronic von Frey, thermal place-preference, and electric foot-shock tests but not in the hot-plate test (*n* = 16 mice [WT-DMSO], 15 [WT-CNO], 15 [HT-DMSO], 15 [HT-CNO] for von Frey, 13, 13, 15, and 12 [thermal place preference], 15, 14, 16, and 14 [hot-plate], 12, 9, 9, and 9 [electric foot-shock], two-way ANOVA with Tukey’s test [von Frey, thermal place-preference], two-way ANOVA [hot-plate], two-way ANOVA with/without Tukey’s test [electric foot-shock; significant genotype x drug interaction for the 2nd but not 1st shock]). Significance is indicated as * (<0.05), ** (<0.01), *** (<0.001), or ns (not significant).

To determine if the neuronal hyperactivity in the *Grin2b*^C456Y/+^ ACC leads to sensory hypersensitivity in *Grin2b*^C456Y/+^ mice, we set out to suppress ACC neuronal activity by a chemogenetic approach using DREADD. *Grin2b*^C456Y/+^ and control (WT) mice were infected with AAV-CaMKIIα-hM4Di-mCherry in the ACC at postnatal week 5, and sensory tests were conducted at postnatal week 8 in the presence of CNO or DMSO (control) (Fig. 4a).

ACC inhibition normalized the somatosensory hypersensitivity of *Grin2b*^C456Y/+^ mice in the electronic von Frey test, without affecting open-field locomotion or anxiety-like behavior (Fig. 4b–f). It also suppressed the thermal hypersensitivity of *Grin2b*^C456Y/+^ mice in the thermal place-preference test, although the hot-plate phenotype was not rescued (Fig. 4g, h). Moreover, ACC inhibition rescued the electric foot-shock phenotype in *Grin2b*^C456Y/+^ mice (Fig. 4i).

In control experiments, CNO or DMSO treatment of *Grin2b*^C456Y/+^ and WT mice injected with control virus (AAV-CaMKIIα-mCherry without hM4Di) had no effect on open-field activity or sensory hypersensitivity (Supplementary Fig. 5a-i). In addition, the concentrations of clozapine, which is a reverse-metabolite of CNO (clozapine-N-oxide) known to actually act on DREADD receptors in the brain [60], were comparable in WT and *Grin2b*^C456Y/+^ brains, as determined by mass spectrometry (Supplementary Fig. 5j). Moreover, inhibition of a non-ACC brain region (S1, somatosensory cortex) did not rescue the sensory hypersensitivity in *Grin2b*^C456Y/+^ mice (Supplementary Fig. 6). Lastly, early postnatal chronic treatment of *Grin2b*^C456Y/+^ mice with D-cycloserine, which could rescue anxiolytic-like behavior in these mice [48], had no effect on sensory hypersensitivity (Supplementary Fig. 7). These results collectively suggest that chemogenetic inhibition of ACC pyramidal neurons normalizes the sensory hypersensitivity of *Grin2b*^C456Y/+^ mice in the von Frey, thermal place-preference, and electric foot-shock tests.

### Chemogenetic ACC inhibition normalizes whisker stimulation-related hyperactivity in the *Grin2b*^C456Y/+^ brain

**Fig. 5:**
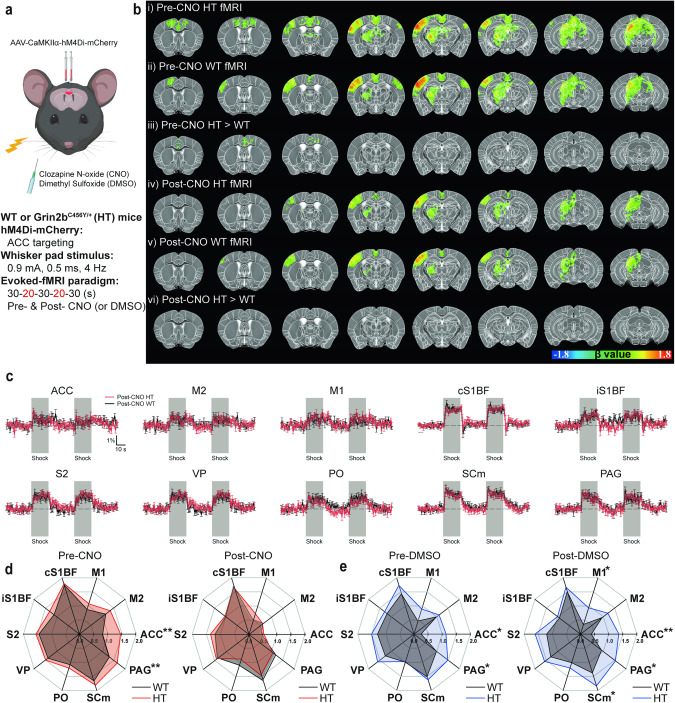
Chemogenetic ACC inhibition normalizes whisker stimulation-related hyperactivity in the *Grin2b*^C456Y/+^ brain. **a** Schema of chemogenetic modulation. Injection of AAV-CaMKIIα-hM4Di-mCherry into the *Grin2b*^C456Y/+^ ACC (5 weeks) was followed by CNO/DMSO treatment and fMRI measurements of brain regional activities (15.2 Tesla) induced by whisker stimulation of mice at 2–3 months. **b** Group fMRI maps in *Grin2b*^C456Y/+^ and WT brains before/after CNO treatment (pre/post-CNO). Note that the pre-CNO HT > WT differences are detectable in the ACC region, although they are less strong than those in Fig. 2f, likely because of variability in virus injection. **c** Examples of whisker stimulation-induced fMRI traces in different brain regions of *Grin2b*^C456Y/+^ and WT mice in the presence of the CNO treatment. Note that stimulus-evoked fMRI signals in ACC and PAG regions of *Grin2b*^C456Y/+^ and WT mice are no longer statistically different after CNO treatment. **d**, **e** CNO treatment normalizes whisker stimulation-induced fMRI signals in ACC and PAG regions of *Grin2b*^C456Y/+^ mice, as shown by comparison of pre/post-CNO fMRI signals in different brain regions (**d**). Note that control treatment (DMSO) does not normalize neuronal hyperactivity in the *Grin2b*^C456Y/+^ ACC or PAG (**e**). Related brain images and fMRI signals traces are not shown, as in those for *Grin2b*^C456Y/+^ mice (**b**, **c**). (*n* = 11 mice [HT-pre/post-CNO], 11 [WT-pre/post-CNO], 7 [HT-pre/post-DMSO], 7 [WT-pre/post-DMSO], Student’s t-test). Significance is indicated as * (<0.05), ** (<0.01), *** (<0.001), or ns (not significant).

As chemogenetic inhibition of the *Grin2b*^C456Y/+^ ACC normalized the behavioral sensory hypersensitivity seen in these mice, we next tested if it could also normalize their neuronal hyperactivity. *Grin2b*^C456Y/+^ and WT mice were infected with AAV-CaMKIIα-hM4Di-mCherry in the ACC followed by fMRI responding to whisker stimulation in the presence of CNO or DMSO (control) (Fig. 5a).

Chemogenetic ACC inhibition normalized the whisker stimulation-induced hyperactivity in the *Grin2b*^C456Y/+^ ACC (Fig. 5b–d). In addition, a decrease was observed in the PAG. Two control experiments were performed. In *Grin2b*^C456Y/+^ and WT mice infected with AAV-CaMKIIα-hM4Di-mCherry in the ACC, DMSO treatment had no effect on the genotype difference in ACC and PAG neurons (Fig. 5e). Additionally, in WT and *Grin2b*^C456Y/+^ mice, whisker stimulation-induced fMRI signals were not affected by CNO treatment (Supplementary Fig. 8), suggesting CNO itself minimally affects whisker stimulation.

These results collectively suggest that chemogenetic inhibition of ACC normalizes the whisker stimulation-induced hyperactivity of ACC and PAG neurons in *Grin2b*^C456Y/+^ mice.

### Chemogenetic ACC inhibition normalizes hyperconnectivity in the *Grin2b*^C456Y/+^ brain

The results described thus far suggest that the hyperactivity of *Grin2b*^C456Y/+^ ACC neurons promotes the sensory hypersensitivity of the mutant mice. Next, we asked: How might the network connectivity in the *Grin2b*^C456Y/+^ brain play a role in the behavioral sensory hypersensitivity of these mice? To this end, we analyzed network connectivity in the *Grin2b*^C456Y/+^ brain under baseline and sensory stimulation conditions based on the correlated activities of different brain regions in the fMRI data.

**Fig. 6:**
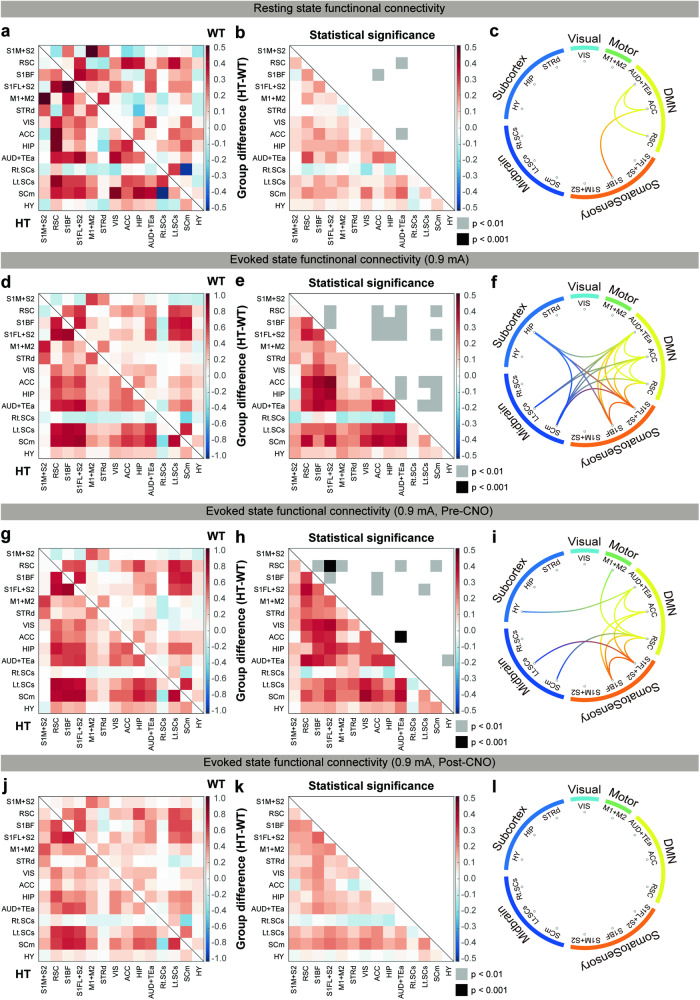
Chemogenetic ACC inhibition normalizes baseline and evoked hyperconnectivity in the *Grin2b*^C456Y/+^ brain. **a**–**c** Brain connectivity matrices obtained under baseline conditions showing correlative fMRI activities in different brain regions of WT and *Grin2b*^C456Y/+^ mice (2–3 months) (**a**), HT-WT differences with *p* values (**b**), and a circular connectivity map based on the results in panel b (**c**). 14 RSN components were used for functional connectivity analyses. S1M + S2 primary somatosensory cortex of mouth and partial secondary somatosensory, RSC retrosplenial cortex, S1BF S1 of barrel field, S1FL + S2 S1 of forelimb and partial S2, M1 + M2 primary motor and secondary motor cortex, STRd striatum dorsal, VIS visual cortex, ACC anterior cingulate cortex, HIP hippocampus, AUD+TEa auditory cortex and temporal association cortex, Rt.SCs right superior colliculus sensory-related, Lt.SCs left superior colliculus sensory-related, SCm superior colliculus motor-related, HY hypothalamus. (*n* = 11 mice [HT], 10 [WT], Student’s t-test). Brain connectivity matrices obtained under whisker stimulation (0.9 mA) conditions showing correlative fMRI activities in WT and *Grin2b*^C456Y/+^ mice (2–3 months) (**d**), HT-WT differences (**e**), and a circular connectivity map (**f**). Note that increased connectivity is also observed in connections involving ACC/non-ACC and cortical/subcortical regions. (*n* = 11 [HT], 10 [WT], Student’s t-test). Brain connectivity matrices obtained under whisker stimulation (0.9 mA) in the absence of chemogenetic inhibition, showing correlative fMRI activities in WT and *Grin2b*^C456Y/+^ mice (2–3 months) (**g**), HT-WT differences (**h**), and a circular connectivity map (**i**) (*n* = 11 [HT-pre-CNO], 11 [WT-pre-CNO], Student’s t-test). **j**–**l** Brain connectivity matrices obtained under whisker stimulation (0.9 mA) in the presence of chemogenetic inhibition, showing correlative fMRI activities in WT and *Grin2b*^C456Y/+^ mice (2–3 months) (**j**), HT-WT differences (**k**), and a circular connectivity map (**l)** (*n* = 11 [HT-post-CNO], 11 [WT-pre-CNO], Student’s t-test). Significance is indicated as * (<0.05), ** (<0.01), *** (<0.001), or ns not significant.

*Grin2b*^C456Y/+^ mice displayed increases in baseline connectivity between the ACC and S1BF as well as within the regions of the default mode network (between the ACC and AUD+TEa and between the ACC and RSC) (Fig. 6a–c), known to be associated with ASD [61–64]. Under whisker stimulation conditions, increased network activity was observed in additional connections, including ACC-cortical connections (ACC-S1FL + S2 and ACC-RSC) and ACC-subcortical connections (ACC-SCs/m) (Fig. 6d–f). In addition, increased connectivity was observed in multiple non-ACC connections, including cortico-cortical and cortico-subcortical connections.

The functional network of *Grin2b*^C456Y/+^ mice injected with AAV-CaMKIIα-hM4Di-mCherry in the ACC was assessed prior to CNO injection, revealing heightened network activity involving the ACC compared with WT mice (Fig. 6g–i). This was similar, although not identical, to the network hyperactivity observed in naïve *Grin2b*^C456Y/+^ mice (Fig. 6d–f). Importantly, CNO treatment eliminated the heightened network activity in *Grin2b*^C456Y/+^ mice (Fig. 6j–l). In control experiments using WT and *Grin2b*^C456Y/+^ mice, connectivity patterns were not affected only by CNO treatment in in either WT or *Grin2b*^C456Y/+^ mice (Supplementary Fig. 9), suggesting CNO itself minimally affects brain connectivity.

These results suggest that *Grin2b* haploinsufficiency leads to baseline hyperconnectivity between the ACC and somatosensory cortex as well as whisker stimulation-induced hyperconnectivity involving ACC/non-ACC and cortical/subcortical regions. In addition, these heightened network activities (baseline and sensory-evoked) are normalized by chemogenetic inhibition of ACC pyramidal neurons, in line with the reported hyperconnectivity of the default mode network in ASD [61–64].

## Discussion

Our study reveals sensory supraspinal mechanism-related hypersensitivity in *Grin2b*^C456Y/+^ mice and hyperactivity in certain brain regions, including ACC. The neuronal hyperactivity of the ACC involves increased excitatory synaptic transmission, and chemogenetic inhibition of ACC neurons reverses the sensory hypersensitivity. Connectivity analyses indicate that there is baseline and evoked hyperconnectivity between ACC and other cortical/subcortical regions, and that this is normalized by chemogenetic ACC inhibition.

*Grin2b*^C456Y/+^ mice show sensory hypersensitivity to mechanical, thermal, and electrical stimuli. These changes involve supra-spinal mechanisms, as supported by the normal sensory functions observed in *Cdx2-Cre;Grin2b*^fl/+^ mice. These results differ from the previous results obtained using mouse models of ASD, highlighting the peripheral roles of *Mecp2* and *Gabarb3*, expressed in peripheral somatosensory neurons, and *Shank3*, expressed in peripheral dorsal root ganglia neurons, in the regulation of sensory functions [12, 14, 16, 21]. Therefore, our results constitute a case where supraspinal but not subspinal mechanisms underlie ASD-related sensory hypersensitivity.

The present c-fos and fMRI results reveal, in an unbiased manner, the brain regions that respond more strongly to sensory stimuli, including the ACC. The ACC is involved in pain processing and perception [57–59], and our study links it with sensory hypersensitivity in a mouse model of ASD, extending the previous studies highlighting the role of the primary somatosensory cortex in sensory hyper/hyposensitivity in mouse models of ASD (*Shank3*- and *Syngap1*-mutant mice) [11, 13].

Our electrophysiology results suggest that increased excitatory synaptic transmission may contribute to the hyperactivity of mutant ACC neurons. How might *Grin2b* deletion promote excitatory synaptic function? GluN2B-containing NMDARs critically regulate long-term depression (LTD) at developing excitatory synapses [39]. Accordingly, *Grin2b*^C456Y/+^ synapses display decreased NMDAR currents and impaired LTD at hippocampal excitatory synapses [48]. Given that LTD in developing brains is known to refine neural circuits by redistributing synaptic proteins to more active synapses [40, 41], impaired LTD in *Grin2b*^C456Y/+^ mice [48] may lead to insufficient circuit refinement and neuronal hyperconnectivity, as supported by the enhanced excitatory synaptic transmission and fMRI hyperconnectivity seen in *Grin2b*^C456Y/+^ mice. Our results, which are in line with the ASD-related hypothesis of excitation-inhibition imbalance [65–68], extend the recent study on *Tsc2*-mutant mice where excessive mTOR signaling causes increased excitatory synaptic density and fMRI-supported brain hyperconnectivity [69]. In addition, our results mainly involving changes in pyramidal neurons are distinct from the previous result that impaired GABA neurons abnormally activate target pyramidal neurons in *Shank3*-mutant mice [11]. Our results also differ from the decreased (not increased) excitatory synaptic transmission found in somatosensory cortical neurons of *Syngap1*-mutant mice [13].

Our connectivity analyses indicate that there is baseline hyperconnectivity between the ACC and S1BF/AUD+TEa and sensory stimulation-induced hyperconnectivity between the ACC and other cortical and subcortical regions in the mutant mice. In addition, chemogenetic inhibition of ACC neurons, which normalizes sensory hypersensitivity, also normalizes these network hyperconnectivities, causally linking the brain hyperconnectivity with sensory hypersensitivity in *Grin2b*^C456Y/+^ mice. The hyperconnectivity in the *Grin2b*^C456Y/+^ brain may involve a hyperconnection between the ACC and S1, which are known to regulate affective and sensory aspects of pain, respectively [70–74]. In line with this hypothesis, S1 input to the ACC promotes nociceptive responses, and chronic pain enhances ACC-S1 connectivity [75]. In addition, ACC-S1 hyperconnectivity involving excitatory synapses promotes both sensory and affective pain responses [76, 77]. Although further details remain to be determined, our study constitutes a platform where ASD-related excessive pain perception and responses [7–10] could be further explored.

In summary, our study shows that *Grin2b* haploinsufficiency in mice leads to behavioral sensory hypersensitivities that are causally associated with hyperactive ACC neurons with increased excitatory synaptic transmission and ACC-related brain regional hyperconnectivity under baseline and evoked states.

### Reporting summary

Further information on research design is available in the Nature Research Reporting Summary linked to this article.

## Supplementary information

Supplementary information

Reporting summary

Source data

Supplementary Table 1

## Data Availability

The source data underlying all figures and Supplementary figures are provided as Source Data files. All data reported in this study are available from the corresponding authors upon request. Source data are provided in this paper.
