# Supplementary material for: Anterior cingulate cortex-related functional hyperconnectivity underlies sensory hypersensitivity in Grin2b-mutant mice
Source: Mol Psychiatry. 2024 May 4;29(10):3195–207. doi: 10.1038/s41380-024-02572-y (PMC11449790; doi:10.1038/s41380-024-02572-y)
Supplement: Supplementary file 1 — Supplementary information [file 41380_2024_2572_MOESM1_ESM.docx]

**Anterior cingulate cortex-related functional hyperconnectivity underlies sensory hypersensitivity in *Grin2b*-mutant mice**

Soowon Lee^1,2,*^, Won Beom Jung^3,4,*^, Heera Moon^5,*^, Geun Ho Im^3^, Young Woo Noh^6^, Wangyong Shin^6^, Yong Gyu Kim^6^, Jee Hyun Yi^6^, Seok Jun Hong^3,7^, Yongwhan Jung^8^, Sunjoo Ahn^8^, Seong-Gi Kim^3,7,9,#^, and Eunjoon Kim^5,6,#^

^1^Graduate School of Medical Science and Engineering, Korea Advanced Institute of Science and Technology (KAIST), Daejeon 34141, Korea; ^2^Department of Anesthesiology and Pain Medicine, Seoul National University Bundang Hospital, Seongnam 13620, Korea; ^3^Center for Neuroscience Imaging Research, Institute for Basic Science (IBS), Suwon 16419, Korea; ^4^Emotion, Cognition & Behavior Research Group, Korea Brain Research Institute (KBRI), Daegu 41062, Korea;

^5^Department of Biological Sciences, Korea Advanced Institute of Science and Technology (KAIST), Daejeon 34141, Korea; ^6^Center for Synaptic Brain Dysfunctions, Institute for Basic Science (IBS), Daejeon 34141, Korea; ^7^Department of Biomedical Engineering, Sungkyunkwan University, Suwon 16419, Korea; ^8^Therapeutics and Biotechnology Division, Korea Research Institute of Chemical Technology (KRICT), Daejeon 34114, Korea; ^9^Department of Intelligent Precision Healthcare Convergence, Sungkyunkwan University, Suwon 16419, Korea; *These authors contributed equally to the work; ^#^Co-corresponding authors

### **Supplementary materials and methods**

### **Electronic von Frey test**

### The mechanical withdrawal threshold was measured using electronic von Frey filaments and a dynamic plantar aesthesiometer (Ugo Basile). The mice were placed individually in small cages on the iron mesh of the apparatus at least 1 hour before the experiment to allow for acclimation to the equipment. A rigid filament was applied with increasing force until a paw withdrawal response was elicited. The withdrawal threshold was automatically recorded by the electronic device.

### **Hot-plate test**

###### The hot-plate test was used to determine the response threshold of a subject mouse to a heat stimulus ^1^. Unrestrained mice were placed on a metal surface maintained at a constant temperature (55 ºC) and the latency to response, meaning the time taken to observe nocifensive behaviors, was recorded by the investigator. Nocifensive behaviors included hind paw withdrawal, shaking, licking, stamping, and jumping ^2^. If the mouse did not show any nocifensive behavior within 60 sec, the test was terminated to avoid tissue damage and the latency to response was recorded as 60 seconds.

### **Thermal place-preference test**

###### The thermal place-preference test was conducted to evaluate the behavioral aversion to a thermal place, as described previously ^3^. Two floors, one set to a room temperature (30 ºC) and one set to a high temperature (40 ºC), were surrounded by opaque plexiglass walls (13 x 3 x 9.5 inch). Mice were allowed to freely explore the two chambers for 10 minutes. The percentage of time spent on the hot floor during the last 5 minutes was measured.

### **Electric foot-shock test**

###### Experiments were carried out in a fear-conditioning system (Coulbourn Instruments). Training and testing were performed in a Plexiglas chamber with a stainless steel grid floor. On the training day, mice were placed in the fear chamber and allowed to freely move around the chamber for 2 minutes before they received two or five foot shocks (2 sec, 0.6 mA, 2 minutes apart). The freezing time of mice after the acute nociceptive stimuli was measured.

### **Open-field test**

###### The open-field test was performed to evaluate locomotor activity and anxiety-like behavior. The apparatus (40 x 40 x 40 cm) consisted of a floor surrounded by four white acryl walls. Mice were allowed to freely explore the apparatus for 1 hour, while the movements were recorded. Locomotor activity and time spent in the center zone (20 x 20 cm) of the apparatus were analyzed using Ethovision XT 10 (Noldus). In the open-field test with chemogenetic manipulation, mice explored the chamber for 15 but not 60 min to evaluate changes in locomotor activity during a time period comparable to those used for other somatosensory behavioral tests.

### **Light-dark test**

###### The light-dark test was performed to evaluate anxiety-like behavior. The apparatus consisted of two chambers (20 x 30 x 20 cm for light chamber; 20 x 13 x 20 cm for dark chamber) with an entrance between the two chambers. Light conditions were 400 lux for the light chamber and 0 lux for the dark chamber, respectively. Mice were allowed to freely explore the two chambers for 10 min. Recorded mouse movements were analyzed using Ethovision XT 10 (Noldus).

### **Elevated plus-maze test**

The elevated plus-maze test was performed to evaluate anxiety-like behavior. The apparatus consists of two open arms (30 x 5 x 0.5 cm), two closed arms (30 x 5 x 30 cm), and a center zone. Mice were allowed to freely explore all areas, located 75 cm above the floor. Light conditions of each chamber were ~200 lux for open arms and ~20 lux for closed arms. Mouse movements recorded for 8 min were analyzed using Ethovision XT 10 (Noldus).

## **Electrophysiology**

###### NMDG-HEPES artificial cerebrospinal fluid (aCSF) consisted of NMDG (100 mM), N-acetylcysteine (NAC, 12mM), NaHCO_3_ (30 mM), HEPES (20 mM), Glucose (25 mM), Thiourea (2 mM), Na-Ascorbate (5 mM), Na-pyruvate (3 mM), KCl (2.5 mM), NaH_2_PO_4_ (1.25 mM), CaCl_2_ (0.5mM), and MgSO_4_ (10 mM). HEPES aCSF consists of NaCl (92 mM), NAC (12 mM), NaHCO_3_ (30 mM), HEPES (20 mM), glucose (25 mM), thiourea (2 mM), Na-ascorbate (5mM), Na-pyruvate (3 mM), KCl (2.5 mM), NaH_2_PO_4_ (1.25 mM), MgCl_2_ (1.3 mM), and CaCl_2_ (2.5 mM). The pH and osmolality of NMDG-HEPES aCSF and HEPES aCSF solutions were adjusted to 7.3~7.4 and 300~310 mOsm/kg, respectively. The aCSF consisted of NaCl (125 mM), NaHCO_3_ (25 mM), glucose (10 mM), KCl (2.5 mM), NaH_2_PO_4_ (1.25 mM), MgCl_2_ (1.3 mM), and CaCl_2_ (2.5 mM). All aCSF solutions were saturated with a mixture of 95% O_2_ and 5% CO_2_ before use.

###### Under isoflurane-based anesthesia, mice were perfused with 30 ml (room temperature) of carbonated NMDG-HEPES aCSF. Brains were extracted from the mice and coronally sliced (300 μm thickness) using vibratome (VT1200s, Leica) in a reservoir filled with oxygenated NMDG-HEPES aCSF. All brain slices were transferred to the initial recovery chamber filled with pre-warmed (32 ºC), oxygenated NMDG-HEPES aCSF for 11 min. For the secondary recovery, all brain slices were transferred to the HEPES aCSF chamber at room temperature for more than 1 hour.

###### Whole-cell patch clamp recording of excitatory neurons in layer 2/3 of the ACC was conducted using MultiClamp 700B amplifier (Molecular Devices) and Digidata 1550 (Molecular Devices). To identify excitatory neurons, we used red-fluorescence protein (RFP) signal from *CaMKII*$\alpha$*-Cre*;Ai9*;Grin2b* ^C456Y/+^ mice. During whole-cell patch clamp recordings, series resistance was monitored each sweep by measuring the peak amplitude of the capacitance currents in response to short hyperpolarizing step pulse (5 mV, 40 ms); only cells with a change in < 20% were included in the analysis. Recording and stimulation pipettes were pulled from borosilicate glass capillaries (Harvard Apparatus) using a micropipettes electrode puller (Narishiege).

###### Whole-cell recordings of mEPSCs were obtained in excitatory neurons in layers 2/3 of ACC at a holding potential of –70 mV. To inhibit spontaneous action potential and ISPCs, tetrodotoxin (TTX; 1 $\mu$M) and picrotoxin (PTX; 100 $\mu$M) were added into aCSF. The recording pipettes (3.0~4.0 MΩ) were filled with an internal solution containing (in mM) 100 CsMeSO_4_, 10 TEA-Cl, 8 NaCl, 10 HEPES, 5 QX-314-Cl, 2 Mg-ATP, 0.3 Na-GTP, and 10 EGTA, with pH 7.25, 295 mOSm. Whole-cell recordings of mIPSCs were obtained in excitatory neurons in layers 2/3 of the ACC at a holding potential of –70 mV. To inhibit spontaneous action potential and ESPCs, tetrodotoxin (TTX; 1 $\mu$M), NBQX (10 $\mu$M), and D-AP5 (50 $\mu$M) were added into aCSF. The recording pipettes (3.0~4.0 M$\Omega$) were filled with an internal solution containing (in mM) 120 CsCl, 10 TEA-Cl, 8 NaCl, 10 HEPES, 5 QX-314-Cl, 4 Mg-ATP, 0.3 Na-GTP, and 10 EGTA, pH 7.35, 280 mOSm. To measure spontaneous EPSCs and IPSCs, TTX was omitted to maintain spontaneous network activities. Whole-cell recordings of eEPSC/eIPSC ratio (evoked E/I ratio) were obtained in excitatory neurons in layers 2/3 of ACC with 20 times of electrical stimulation at every 15 s interval. To block NMDAR-mediated EPSCs, D-AP5 (50 $\mu$M) was added into aCSF. The recording pipettes (3.0~4.0 M$\Omega$) were filled with an internal solution containing (in mM) 100 CsMeSO_4_, 10 TEA-Cl, 8 NaCl, 10 HEPES, 5 QX-314-Cl, 2 Mg-ATP, 0.3 Na-GTP, and 10 EGTA, pH 7.25, 295 mOsm. The excitatory neurons in layers 2/3 of ACC were voltage-clamped at –70 mV for eEPSC and 0 mV for eIPSC.

The evoked E/I ratio was determined by dividing the mean value of 20 AMPAR-mediated EPSC peak amplitudes by the mean value of 20 GABAR-mediated IPSC peak amplitudes. Whole-cell current-clamp recordings were performed using the recording pipettes (3.5-5.5 MΩ) filled with an internal solution containing the following (in mM): 135 K-gluconate, 7 NaCl, 10 HEPES, 0.5 EGTA, 2 Mg-ATP, 0.3 Na-GTP, and 10 phosphocreatine, pH 7.3, 295 mOsm. All current-clamp recordings were conducted within the aCSF containing 100 μM PTX, 10 μM NBQX, and 50 μM D-AP5 to block inhibitory and excitatory synaptic inputs. To measure the intrinsic excitability of excitatory neurons in layers 2/3 of ACC, depolarizing currents with a length of 500 ms were injected into neurons from +50 to +500 pA with increments of 50 pA. The amplitude of post-burst afterhyperpolarization (PB-AHP) was measured as the difference between the baseline before the current injection and the negative peak potential following the spiking response of neuron. Input resistance was determined by measuring the difference between the baseline and the maximal negative voltage during hyperpolarizing current injection (from -300 to -50 pA with increments of 50 pA) of 500 msec duration. For the analysis of action potential (AP), the first AP that fired during the injection of depolarizing current was chosen. AP threshold was defined by measuring the membrane potential at which its first derivatives exceeded 5 mV/ms. The differences between the AP threshold and the positive and the negative peak of the trace were defined as the AP amplitude and the AHP amplitude, respectively. Full width at half maximum (FWHM) was the duration of an AP measured between two points on the y-axis at half of the AP amplitude. All current-clamp recording data were analyzed using IntrinsicVIEW Analysis Pack (<https://github.com/parkgilbong/IntrinsicVIEW>).

**References for supplementary methods**

1. WOOLFE G, MACDONALD AD. THE EVALUATION OF THE ANALGESIC ACTION OF PETHIDINE HYDROCHLORIDE (DEMEROL). *Journal of Pharmacology and Experimental Therapeutics* 1944; **80**(3)**:** 300-307.

2. Espejo E. Structure of the rat's behaviour in the hot plate test. *Behavioural Brain Research* 1993; **56**(2)**:** 171-176.

3. Smith ML, Asada N, Malenka RC. Anterior cingulate inputs to nucleus accumbens control the social transfer of pain and analgesia. *Science* 2021; **371**(6525)**:** 153-159.

**Supplementary figure legends**

**
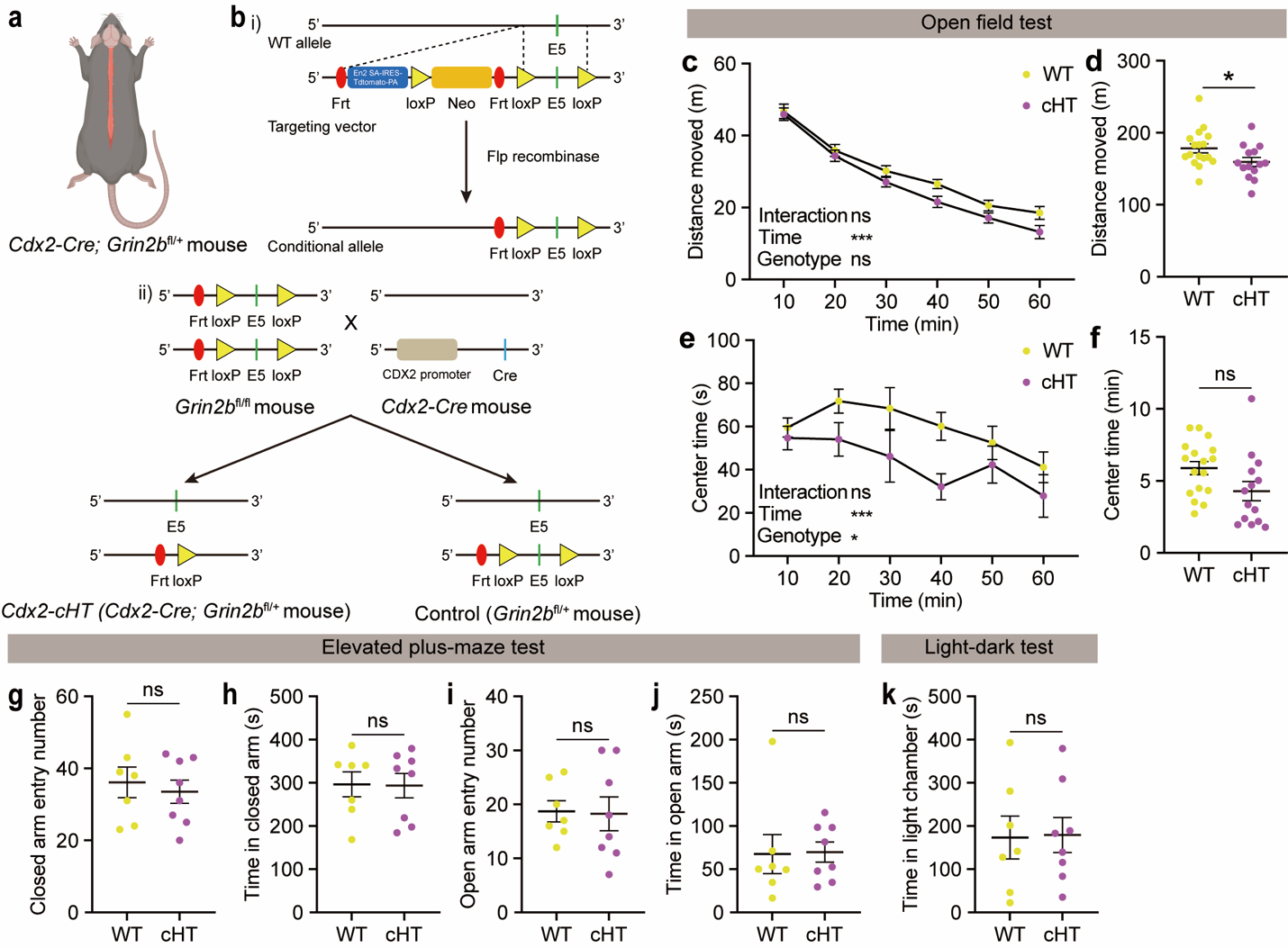
**

**Supplementary Figure 1. *Cdx2-Cre;Grin2b*^fl/+^ mice display moderate hypoactivity without changes in anxiety-like behavior.**

(a) Schematic diagram showing a conditional *Grin2b* deletion restricted to non-brain peripheral regions below the second cervical spinal cord segment.

(b) Conditional *Grin2b* knockout (cKO) strategy by exon 5 (E5) targeting and breeding scheme used to produce *Cdx2-Cre;Grin2b*^fl/+^ and control (*Grin2b*^fl/+^) mice.

(c–f) *Cdx2-Cre;Grin2b*^fl/+^ mice (2–3 months; exon 5 deletion) display moderate hypoactivity in the open-field test, as shown by distance moved, but exhibit normal anxiety-like behavior, as shown by time spent in the center region of the open-field arena. (n = 17 mice [*Grin2b*^fl/+^], 14 [Cdx2-Cre;*Grin2b*^fl/+^], two-way -ANOVA [distance moved; time in center], Student’s t-test [total distance moved; total center time]).

(g–j) Normal anxiety-like behavior of *Cdx2-Cre;Grin2b*^fl/+^ mice (2–3 months) in the elevated plus-maze test, as shown by open/closed-arm time/entry. (n = 7 [*Grin2b*^fl/+^], 8 [Cdx2-Cre;*Grin2b*^fl/+^], Student’s t-test [closed-arm time, open/closed-arm entry], Mann-Whitney test [open-arm time]).

(k) Normal anxiety-like behavior of *Cdx2-Cre;Grin2b*^fl/+^ mice (2–3 months) in the light-dark test, as shown by time in light chamber. (n = 7 [*Grin2b*^fl/+^], 8 [Cdx2-Cre;*Grin2b*^fl/+^], Student’s t-test).

Significance is indicated as * (< 0.05), ** (< 0.01), *** (< 0.001), or ns (not significant).


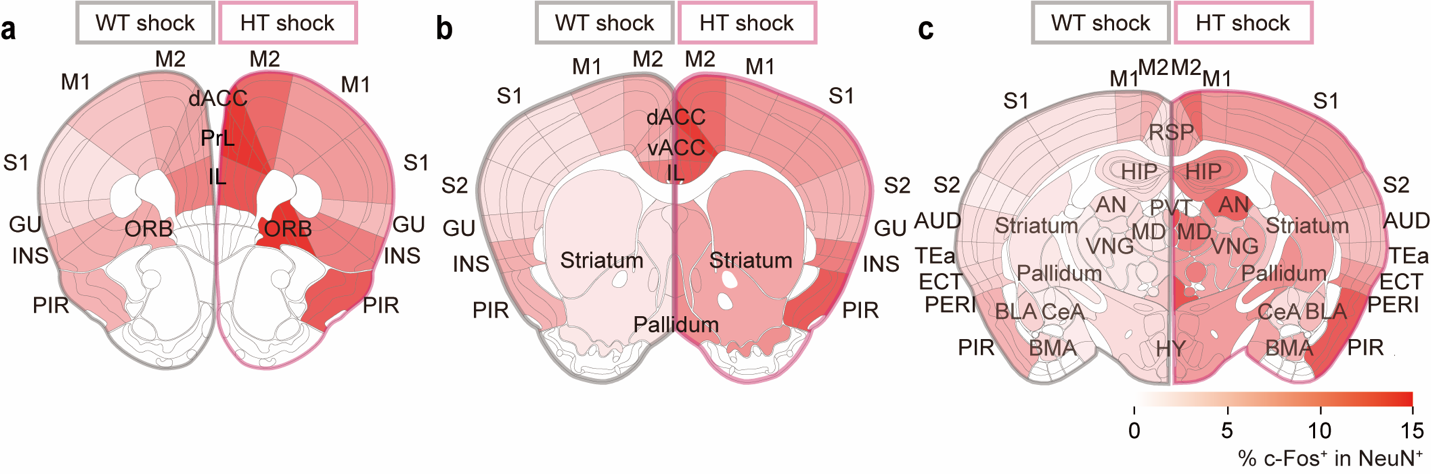


**Supplementary Figure 2. Stronger foot shock-induced increases in c-fos signals in *Grin2b*^C456Y/+^ brain regions, as compared with those in WT mice.**

(a–c) Stronger foot shock-induced increases in c-fos signals in *Grin2b*^C456Y/+^ brain regions, as compared with those in WT mice. ACC, anterior cingulate cortex; AN Thal, anterior group of the dorsal thalamus; BLA, basolateral amygdala; BNST, bed nuclei of the stria terminalis; CeA, central amygdala; ENT, entorhinal cortex; HIP, hippocampus; HY, hypothalamus; IL, infralimbic cortex; IL Thal, intralaminar nuclei of the dorsal thalamus; INS, insular cortex; L Hb, lateral habenula; MD Thal, medial group of the dorsal thalamus; M1, primary motor cortex; M2, secondary motor cortex; ORB, orbital cortex; PAG, periaqueductal gray; PrL, prelimbic cortex; PV Thal, paraventricular nucleus of the thalamus; SCs, superior colliculus; S1, primary somatosensory cortex; S2, secondary somatosensory cortex; VNG Thal, ventral group of the dorsal thalamus.

**
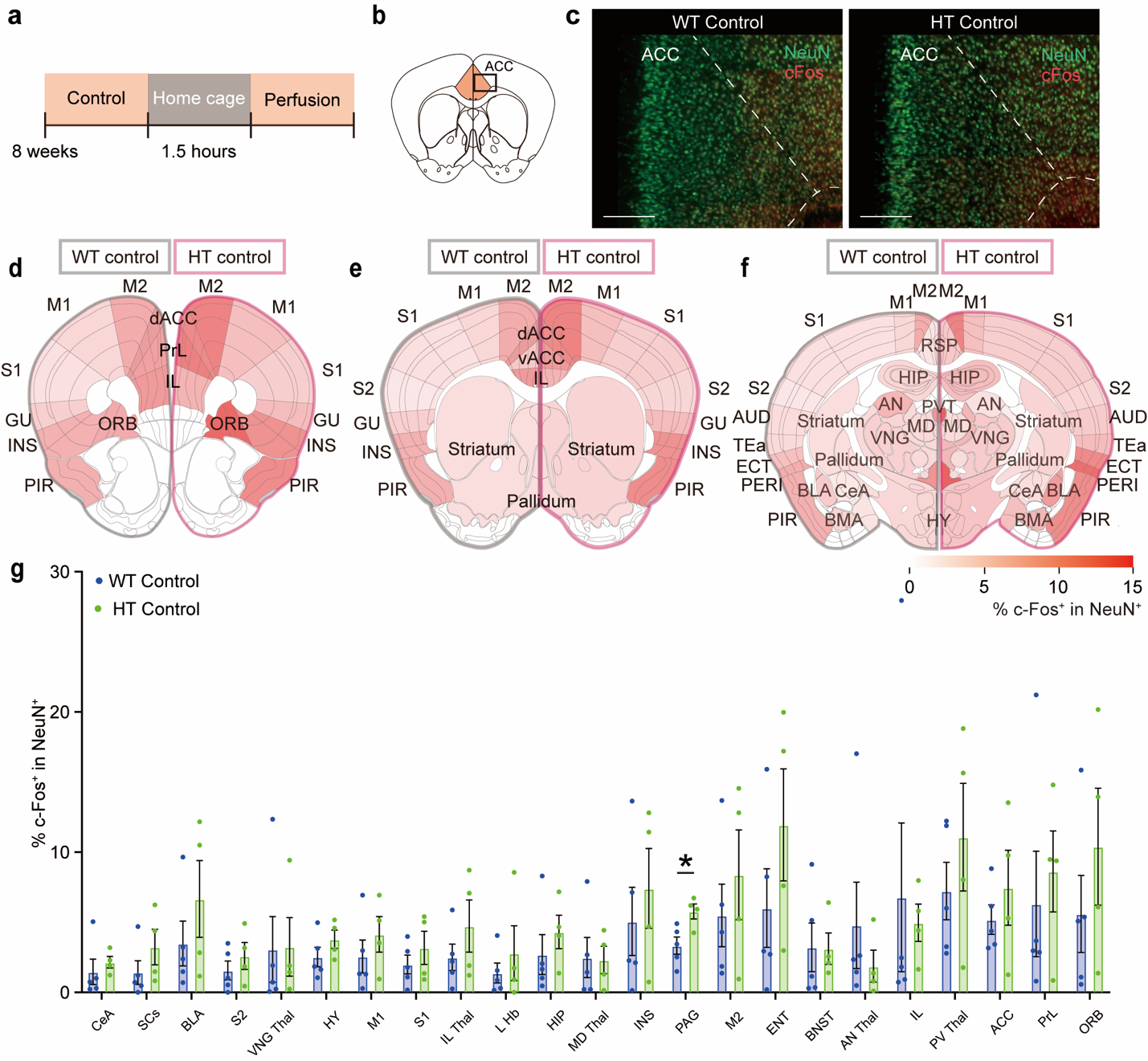
**

**Supplementary Figure 3. *Grin2b*^C456Y/+^ and WT brain regions show largely comparable baseline c-fos signals.**

(a) A schematic diagram showing protocol wherein *Grin2b*^C456Y/+^ mice are exposed to the same shock chamber without electric foot shock, followed by home-cage rest (1.5 hours) and brain perfusion for c-fos staining.

(b and c) Anterior cingulate cortex/ACC as an example of a brain region showing comparable baseline c-fos signals *Grin2b*^C456Y/+^ and WT mice. NeuN staining was performed to identify c-fos signals in neurons. Scale bar, 200 µm.

(d–f) Largely comparable baseline c-fos signals in WT and *Grin2b*^C456Y/+^ brain regions. ACC, anterior cingulate cortex; AN Thal, anterior group of the dorsal thalamus; BLA, basolateral amygdala; BNST, bed nuclei of the stria terminalis; CeA, central amygdala; ENT, entorhinal cortex; HIP, hippocampus; HY, hypothalamus; IL, infralimbic cortex; IL Thal, intralaminar nuclei of the dorsal thalamus; INS, insular cortex; L Hb, lateral habenula; MD Thal, medial group of the dorsal thalamus; M1, primary motor cortex; M2, secondary motor cortex; ORB, orbital cortex; PAG, periaqueductal gray; PrL, prelimbic cortex; PV Thal, paraventricular nucleus of the thalamus; SCs, superior colliculus; S1, primary somatosensory cortex; S2, secondary somatosensory cortex; VNG Thal, ventral group of the dorsal thalamus.

(g) Quantification of the results shown in (d–f). (n = 5 mice [WT], 4 [HT], Student’s t-test).

Significance is indicated as * (< 0.05), ** (< 0.01), *** (< 0.001), or ns (not significant).


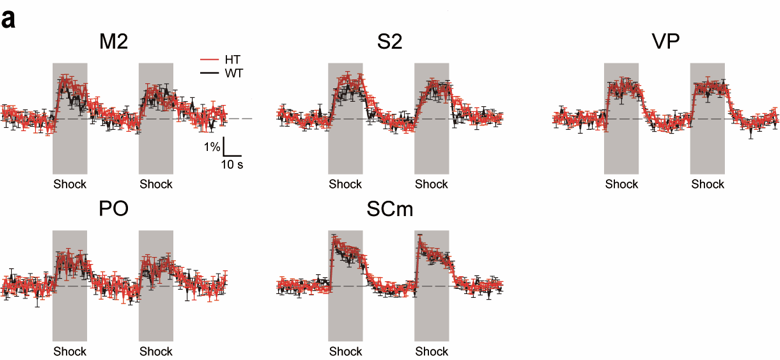


**Supplementary Figure 4. Traces of fMRI signals before/during (grey blocks)/after whisker stimulation.**

(a) Traces of fMRI signals before/during (grey blocks)/after whisker stimulation in different brain regions of *Grin2b*^C456Y/+^ and WT mice (2–3 months). M2, secondary motor cortex; PO, posterior complex of the thalamus; SCm, superior colliculus, motor related; S2, secondary somatosensory cortex; VP, ventral posterior complex of the thalamus.

**
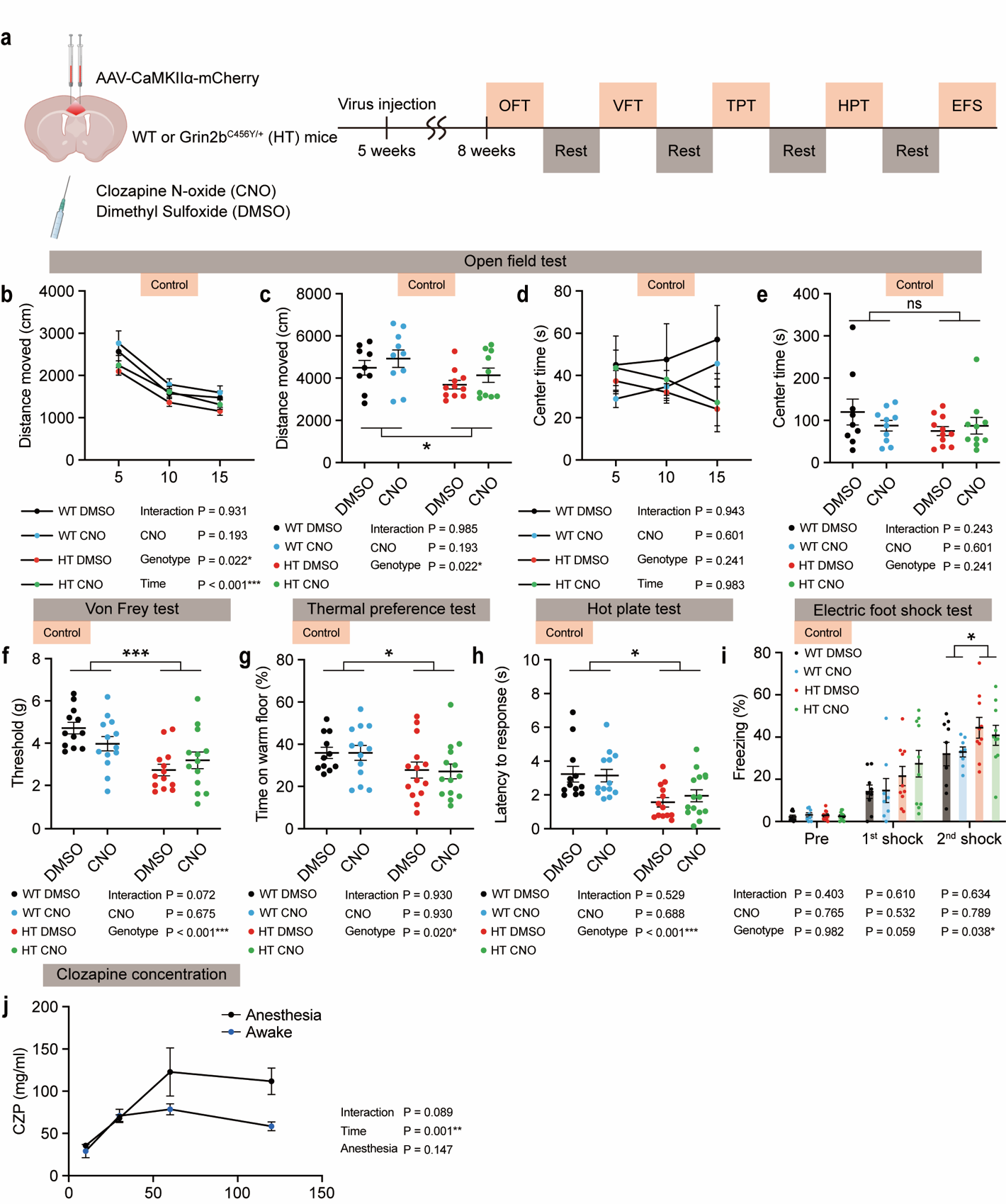
**

**Supplementary Figure 5. Control AAV does not affect locomotion, anxiety-like behavior, or sensory functions of WT or *Grin2b*^C456Y/+^ mice.**

(a) Schema of chemogenetic or DREADD modulation. Injection of AAV-CaMKIIα-mCherry (without h4MDi) into the WT and *Grin2b*^C456Y/+^ ACC was followed by CNO/DMSO treatment and locomotor and sensory tests.

(b–e) Control virus injection and CNO treatment do not affect the open-field locomotion and anxiety-like behavior (center time) of WT and *Grin2b*^C456Y/+^ mice (2–3 months). (n = 9 mice [WT-DMSO], 10 [WT-CNO], 11 [HT-DMSO], 10 [HT-CNO], three-way ANOVA [distance moved/center time], two-way ANOVA [total distance moved/center time])

(f–i) Control virus injection and CNO treatment do not affect the results obtained from WT and *Grin2b*^C456Y/+^ mice (2–3 months) on the electronic von Frey, thermal place-preference, hot-plate, and electric foot-shock tests. (electronic von Frey: n = 12 mice [WT-DMSO], 13 [WT-CNO], 13 [HT-DMSO], 13 [HT-CNO], two-way ANOVA; thermal place-preference: n = 11 mice [WT-DMSO], 13 [WT-CNO], 14 [HT-DMSO], 14 [HT-CNO], two-way ANOVA; hot-plate: n = 12 [WT-DMSO], 13 [WT-CNO], 13 [HT-DMSO], 14 [HT-CNO], two-way ANOVA; electric foot-shock: n = 9 [WT-DMSO], 8 [WT-CNO], 10 [HT-DMSO], 10 [HT-CNO], two-way ANOVA).

(j) Levels of clozapine, a reverse-metabolite of CNO, are comparable in the brains of WT and *Grin2b*^C456Y/+^ mice (2 months), as determined by mass spectrometry. (n = 3 mice [awake-10/30/60/120 min], 3 [anesthesia-60 min], 2 [anesthesia-10/30/120 min; 1 outlying mouse data removed], two-way ANOVA).

Significance is indicated as * (< 0.05), ** (< 0.01), *** (< 0.001), or ns (not significant).


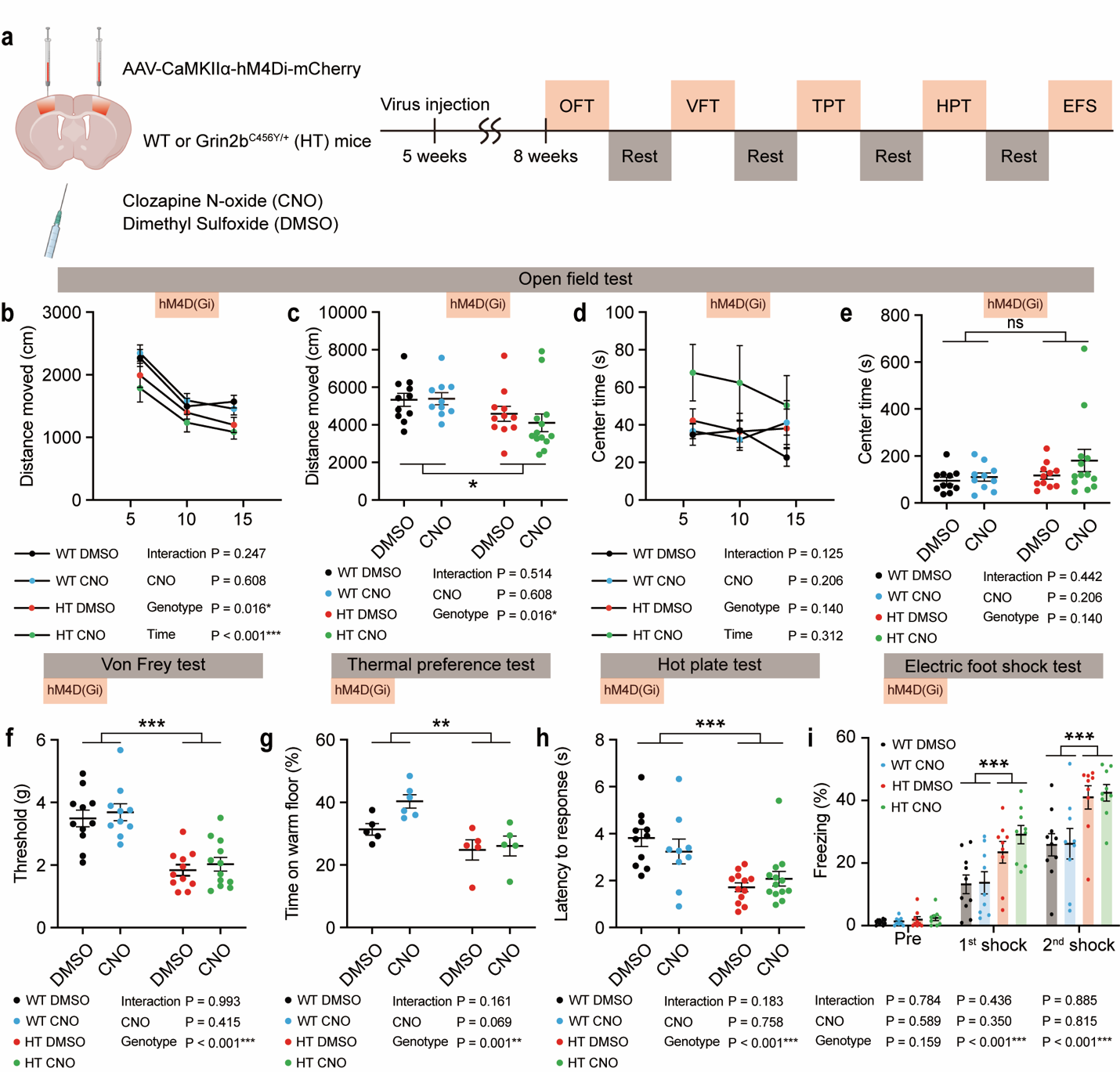


**Supplementary Figure 6. Chemogenetic inhibition of the primary sensory cortex does not normalize sensory hypersensitivity in *Grin2b*^C456Y/+^ mice.**

(a) Schema of chemogenetic modulation using DREADD. Injection of AAV-CaMKIIα-hM4Di-mCherry into the primary sensory cortex (S1) in WT and *Grin2b*^C456Y/+^ mice was followed by locomotor and sensory tests with CNO/DMSO treatment. OFT, open-field test; VFT, von Frey test; TPT, thermal place-preference test; HPT, hot-plate test; EFS, electric foot shock.

(b–e) Chemogenetic inhibition of S1 neurons does not alter open-field locomotion and anxiety-like behavior (center time) in *Grin2b*^C456Y/+^ mice (2–3 months). (n = 11 mice [WT-DMSO], 10 [WT-CNO], 11 [HT-DMSO], 13 [HT-CNO], three-way ANOVA [distance moved/center time], two-way ANOVA [total distance moved/center time]).

(f–i) Chemogenetic inhibition of S1 neurons does not normalize sensory hypersensitivity of *Grin2b*^C456Y/+^ mice (2–3 months) in the electronic von Frey, thermal place-preference, hot-plate, and electric foot-shock tests. (n = 11 mice [WT-DMSO], 10 [WT-CNO], 11 [HT-DMSO], 12 [HT-CNO] for von Frey, 5, 6, 5, and 5 [thermal place-preference], 11, 9, 12, and 13 [hot-plate], 10, 9, 9, and 9 [electric foot-shock], two-way ANOVA [von Frey, thermal place-preference, hot-plate, and electric foot-shock].

Significance is indicated as * (< 0.05), ** (< 0.01), *** (< 0.001), or ns (not significant).


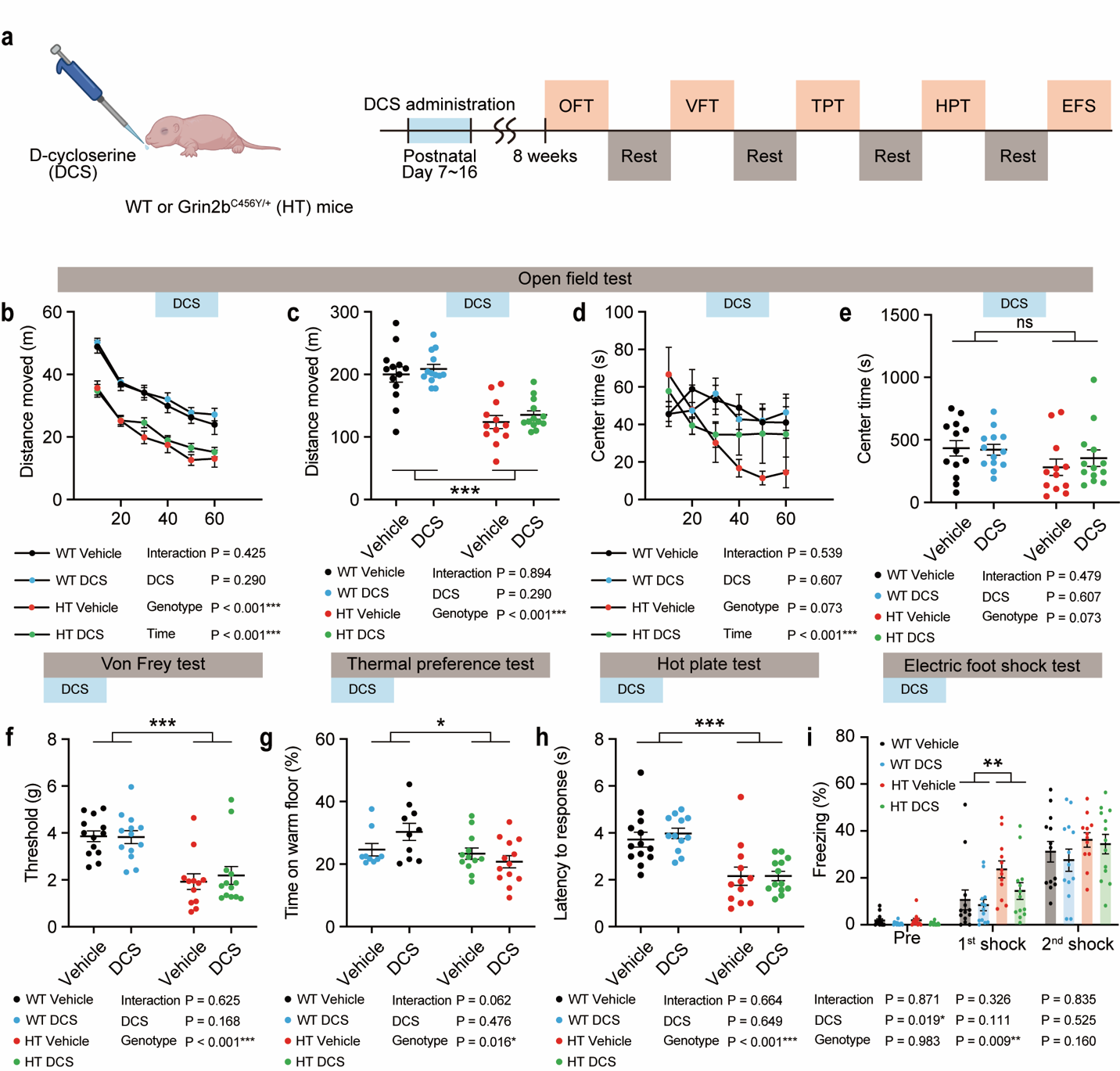


**Supplementary Figure 7. Early chronic D-cycloserine administration does not normalize sensory hypersensitivity in *Grin2b*^C456Y/+^ mice.**

(a) Schema of early postnatal, chronic (P7–16) treatment of WT and *Grin2b*^C456Y/+^ mice with the NMDA receptor agonist D-cycloserine/DCS was followed by locomotor and sensory tests in adult mice (2–3 months). OFT, open-field test; VFT, von Frey test; TPT, thermal place-preference test; HPT, hot-plate test; EFS, electric foot shock.

(b–e) Early chronic DCS treatment (P7–16; twice a day; oral; 40 mg/kg) does not alter open-field locomotion and anxiety-like behavior (center time) in *Grin2b*^C456Y/+^ mice (2–3 months). (n = 13 mice [WT-Veh], 13 [WT-DCS], 12 [HT-Veh], 13 [HT-DCS], three-way ANOVA [distance moved/center time], two-way ANOVA [total distance moved/center time]).

(f–i) Early chronic DCS treatment (P7–16; twice a day; oral; 40 mg/kg) does not normalize sensory hypersensitivity of *Grin2b*^C456Y/+^ mice (2–3 months) in the electronic von Frey, thermal place-preference, hot-plate, and electric foot-shock tests. (n = 13 mice [WT-Veh], 13 [WT-DCS], 12 [HT-Veh], 13 [HT-DCS] for von Frey, 9, 10, 12, and 13 [thermal place preference], 13, 12, 12, and 13 [hot-plate], 13, 13, 12, and 13 [electric foot-shock], two-way ANOVA [von Frey, thermal place-preference, hot-plate and electric foot-shock].

Significance is indicated as * (< 0.05), ** (< 0.01), *** (< 0.001), or ns (not significant).


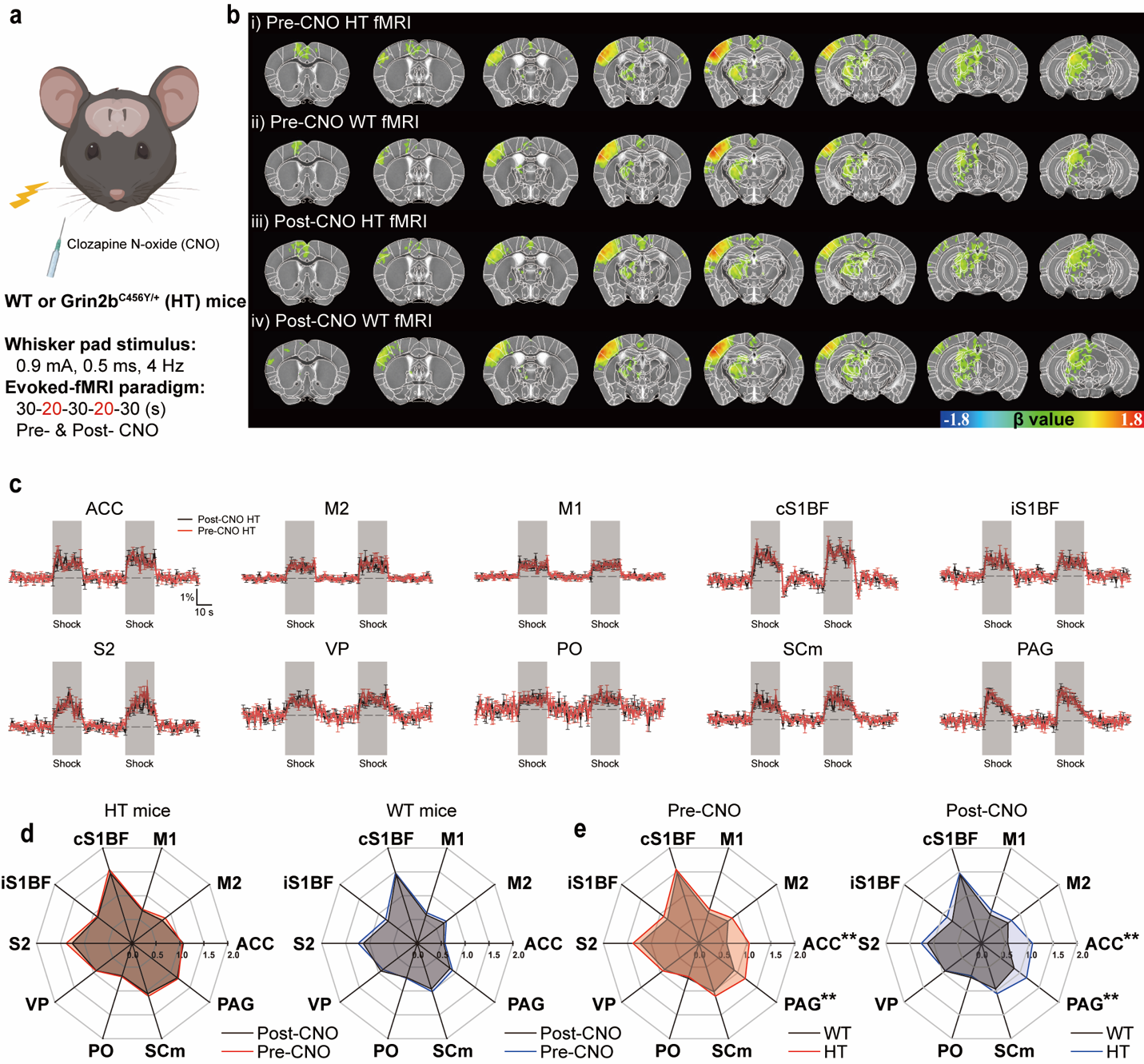


**Supplementary Figure 8. CNO-only injection does not normalize whisker stimulation-related hyperactivity in the *Grin2b*^C456Y/+^ brain**.

(a) Schema of CNO-only injection. fMRI measurements of brain region activity (15.2 Tesla) induced by whisker stimulation of WT and *Grin2b*^C456Y/+^ mice (2–3 months) in the absence of virus injection were performed before and after CNO treatment.

(b) Group fMRI maps in *Grin2b*^C456Y/+^ and WT brains before/after CNO-only treatment (pre/post-CNO).

(c) Examples of whisker stimulation-induced fMRI traces in different brain regions of *Grin2b*^C456Y/+^ mice before and after CNO treatment. Note that stimulus-evoked fMRI signals in various brain regions of *Grin2b*^C456Y/+^ mice are not statistically different before and after CNO treatment.

(d and e) CNO treatment does not affect neuronal activity in the various brain regions of *Grin2b*^C456Y/+^ and WT mice (d). Note that CNO treatment does not normalize whisker stimulation-induced fMRI signals in the ACC and PAG region of the *Grin2b*^C456Y/+^ brain, as shown by comparison of pre/post-CNO fMRI signals in different brain regions (e). Related brain images and fMRI signals traces are not shown, as in those for WT mice (b and c). (n = 9 mice [HT-pre-CNO], 9 [WT-pre-CNO], 9 [HT-post-CNO], 9 [WT-post-CNO], Student’s t-test).

Significance is indicated as * (< 0.05), ** (< 0.01), *** (< 0.001), or ns (not significant).


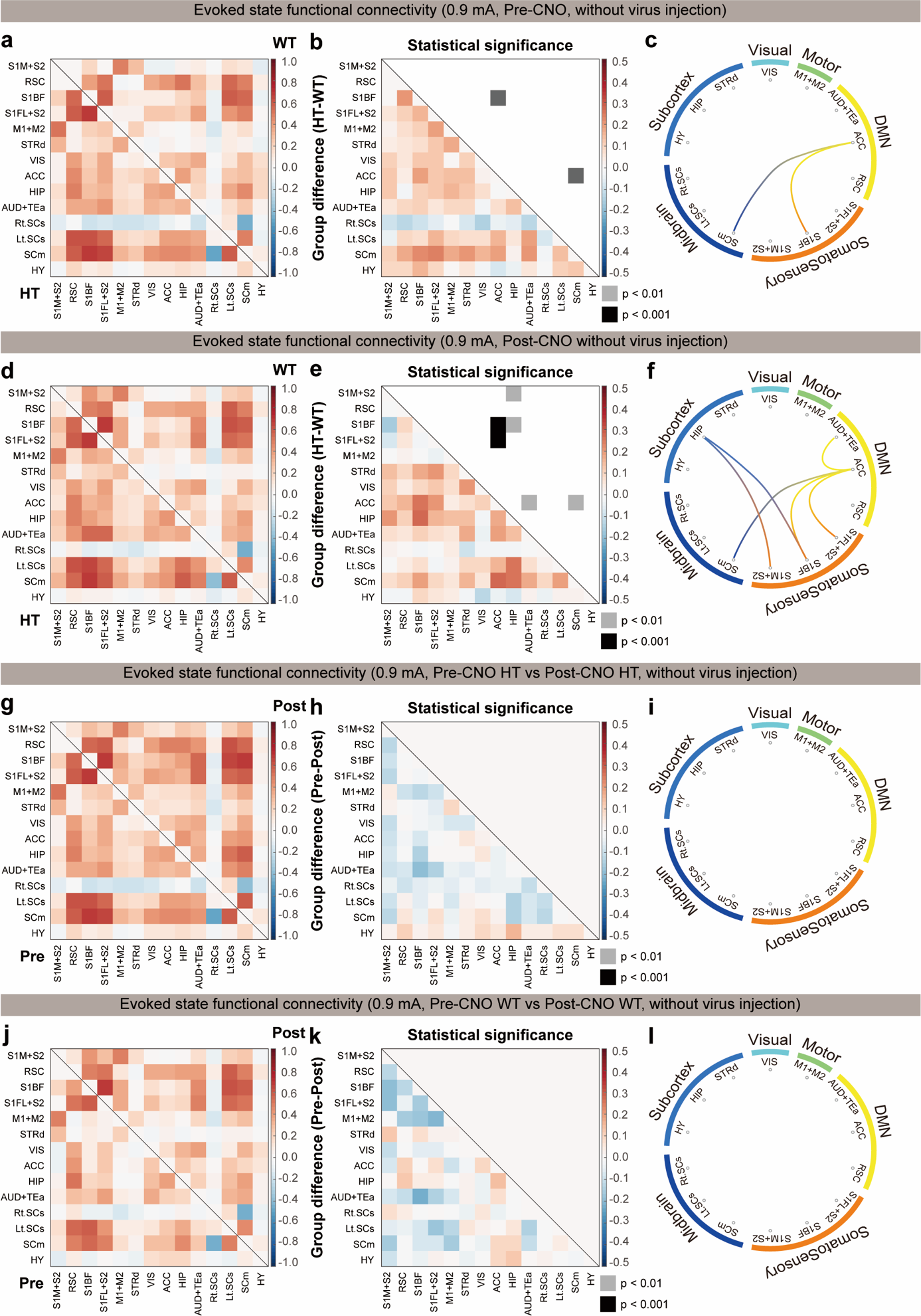


**Supplementary Figure 9. CNO-only injection does not normalize evoked hyperconnectivity in the *Grin2b*^C456Y/+^ brain**.

(a–c) Brain connectivity matrices in WT and *Grin2b*^C456Y/+^ mice (2–3 months) obtained under whisker stimulation (0.9 mA) in the absence of CNO administration, showing correlative fMRI activities (a), HT-WT differences (b), and a circular connectivity (c). Note that increased connectivity is also observed in connections involving ACC and cortical/midbrain regions. ACC, anterior cingulate cortex; M1, primary motor cortex; M2, secondary motor cortex; PAG, periaqueductal gray; PO, posterior complex of the thalamus; SCm, superior colliculus, motor related; cS1, contralateral primary somatosensory cortex; iS1, ipsilateral primary somatosensory cortex; S2, secondary somatosensory cortex; VP, ventral posterior complex of the thalamus. (n = 9 mice [HT], 9 [WT], Student’s t-test).

(d–f) Brain connectivity matrices in WT and *Grin2b*^C456Y/+^ mice (2–3 months) obtained under whisker stimulation (0.9 mA) in the presence of CNO administration, showing correlative fMRI activities (d), HT-WT differences (e), and a circular connectivity map (f). Note that increased connectivity is also observed in connections involving ACC/non-ACC and cortical/midbrain/subcortical regions. (n = 9 [HT], 9 [WT], Student’s t-test).

(g–i) Brain connectivity matrices in *Grin2b*^C456Y/+^ mice obtained under whisker stimulation (0.9 mA) before and after CNO administration, showing correlative fMRI activities (2–3 months) (g), pre-post differences (h), and a circular connectivity map (i). (n = 9 [HT-pre-CNO], 9 [HT-post-CNO], Student’s t-test).

(j–l) Brain connectivity matrices in WT mice (2–3 months) obtained under whisker stimulation (0.9 mA) before and after CNO administration, showing correlative fMRI activities (j), Pre-Post differences (k), and a circular connectivity map (l). (n = 9 [WT-pre-CNO], 9 [WT-post-CNO], Student’s t-test).

Significance is indicated as * (< 0.05), ** (< 0.01), *** (< 0.001), or ns (not significant).

**Supplementary Tables**

**Table S1. Statistical details.**
